# RRM adjacent *TARDBP* mutations disrupt RNA binding and enhance TDP-43 proteinopathy

**DOI:** 10.1093/brain/awz313

**Published:** 2019-10-11

**Authors:** Han-Jou Chen, Simon D Topp, Ho Sang Hui, Elsa Zacco, Malvika Katarya, Conor McLoughlin, Andrew King, Bradley N Smith, Claire Troakes, Annalisa Pastore, Christopher E Shaw

**Affiliations:** 1 United Kingdom Dementia Research Institute Centre, Maurice Wohl Clinical Neuroscience Institute, Institute of Psychiatry, Psychology and Neuroscience, King’s College London, 125 Coldharbour Lane, Camberwell, SE5 9NU, London, UK; 2 York Biomedical Research Institute, Department of Biology, University of York, Wentworth Way, YO10 5DD, York, UK; 3 MRC London Neurodegenerative Diseases Brain Bank, De Crespigny Park, SE5 8AF, London, UK; 4 Centre for Brain Research, University of Auckland, Auckland, New Zealand

**Keywords:** TDP-43, ALS, RNA binding protein, neurodegeneration, protein aggregation

## Abstract

Amyotrophic lateral sclerosis (ALS) presents with focal muscle weakness due to motor neuron degeneration that becomes generalized, leading to death from respiratory failure within 3–5 years from symptom onset. Despite the heterogeneity of aetiology, TDP-43 proteinopathy is a common pathological feature that is observed in >95% of ALS and tau-negative frontotemporal dementia (FTD) cases. TDP-43 is a DNA/RNA-binding protein that in ALS and FTD translocates from being predominantly nuclear to form detergent-resistant, hyperphosphorylated aggregates in the cytoplasm of affected neurons and glia. Mutations in *TARDBP* account for 1–4% of all ALS cases and almost all arise in the low complexity C-terminal domain that does not affect RNA binding and processing. Here we report an ALS/FTD kindred with a novel K181E TDP-43 mutation that is located in close proximity to the RRM1 domain. To offer predictive gene testing to at-risk family members, we undertook a series of functional studies to characterize the properties of the mutation. Spectroscopy studies of the K181E protein revealed no evidence of significant misfolding. Although it is unable to bind to or splice RNA, it forms abundant aggregates in transfected cells. We extended our study to include other ALS-linked mutations adjacent to the RRM domains that also disrupt RNA binding and greatly enhance TDP-43 aggregation, forming detergent-resistant and hyperphosphorylated inclusions. Lastly, we demonstrate that K181E binds to, and sequesters, wild-type TDP-43 within nuclear and cytoplasmic inclusions. Thus, we demonstrate that TDP-43 mutations that disrupt RNA binding greatly enhance aggregation and are likely to be pathogenic as they promote wild-type TDP-43 to mislocalize and aggregate acting in a dominant-negative manner. This study highlights the importance of RNA binding to maintain TDP-43 solubility and the role of TDP-43 aggregation in disease pathogenesis.

## Teaser

TDP-43 is a DNA/RNA binding protein, but whether its interactions with RNA are relevant to inclusion formation in ALS is unclear. Chen *et al.* report that mutated forms of TDP-43 that are unable to bind RNA have an increased tendency to aggregate, and can mediate toxicity by sequestering wild-type TDP-43.

## Introduction

Amyotrophic lateral sclerosis (ALS), also known as motor neuron disease (MND), is the most common adult-onset degenerative disease of motor neurons. The disease is characterized by progressive motor neuron loss from the spinal cord, brainstem and motor cortex, which subsequently leads to muscle weakness and eventual respiratory failure. The average age of disease onset is 55 years. Due to its rapid progression, patients typically die within 3 to 5 years of diagnosis. Riluzole remains the only licensed treatment of ALS in the UK, but only marginally extends life expectancy by a few months (Johnston *et al.*, 2006; Wijesekera and Leigh, 2009; Goyal and Mozaffar, 2014). The cause of ALS is not yet completely defined. The majority of ALS patients have no apparent family history of the disease and are categorized as sporadic, whereas ∼10% of cases are familial. Regardless of the cause, ∼95% of patients share a common molecular pathology featuring the accumulation of ubiquitinated, hyperphosphorylated and detergent resistant TDP-43 protein aggregates in the cytoplasm of affected neuronal tissues (Neumann *et al.*, 2006; Mackenzie *et al.*, 2007; Tan *et al.*, 2007; Geser *et al.*, 2008; Pamphlett *et al.*, 2009; Brettschneider *et al.*, 2013). In 2006, the accumulation of detergent-resistant, hyper-phosphorylated TDP-43 was reported in affected neuronal tissues of ALS and frontotemporal dementia (FTD) cases (Neumann *et al.*, 2006). Shortly afterwards, mutations in TDP-43 were found in patients of both familial and sporadic ALS (Sreedharan *et al.*, 2008). To date, there are more than 50 mutations in TDP-43 reported to be associated with ALS or FTD (for a review see Buratti, 2015) that are estimated to account for ∼4% of familial ALS and ∼1.5% of sporadic ALS (Mackenzie *et al.*, 2010). As TDP-43 cytoplasmic aggregates are observed in ∼95% of ALS and tau-negative FTD, the understanding of the mechanisms contributing to the build-up of TDP-43 proteinopathy is critical in unravelling disease development and holds the key to developing effective therapeutic strategies.

TDP-43 is a DNA and RNA binding protein predominantly localizing in the nucleus but also shuttling between the nucleus and cytoplasm. The architecture of TDP-43 comprises an N-terminal domain, two RRM RNA-binding motifs and a C-terminal region often referred to as the ‘prion-like’ domain, which has low complexity and is enriched for glycine and serine residues. TDP-43 plays a key role in regulating RNA transcription, editing, transport and translation, and is involved in the formation of cellular stress-induced stress granules in the cytoplasm (Belly *et al.*, 2005; Rutherford *et al.*, 2008; Colombrita *et al.*, 2009; Dion *et al.*, 2009; Nonaka *et al.*, 2009). TDP-43 is essential for organism development and survival (Kraemer *et al.*, 2010); however, an excessive amount of the protein causes TDP-43 accumulation, which leads to cytotoxicity and motor deficits (Ash *et al.*, 2010; Barmada *et al.*, 2010; Stallings *et al.*, 2010; Wils *et al.*, 2010). Therefore, it is not surprising that the cellular levels of TDP-43 are under tight control through a system of autoregulation (Ayala *et al.*, 2011; Polymenidou *et al.*, 2011).

The majority of the TDP-43 disease-causing mutations are in the C-terminal region of the protein. We and others have shown that the C-terminal mutations promote TDP-43 translocation to the cytosol, enhance protein stability and promote the aggregation of the mutant protein which in turn leads to enhanced cytotoxicity *in vitro* and *in vivo* (Johnson *et al.*, 2009; Barmada *et al.*, 2010; Ling *et al.*, 2010; Bilican *et al.*, 2012; Mitchell *et al.*, 2015). As a DNA/RNA-binding protein, TDP-43 plays an important role in RNA regulation and transportation (Colombrita *et al.*, 2009; Lagier-Tourenne *et al.*, 2010). However, the role of RNA binding in the build-up of TDP-43 proteinopathy has never been investigated. In this study, we report a novel FTD/ALS-linked mutation, K181E, which causes catastrophic disruption in TDP-43 protein-RNA binding. Investigating the impact of loss of RNA-binding on TDP-43 proteinopathy further, we demonstrate that mutations that leave TDP-43 incapable of binding RNA promote the accumulation of insoluble aggregates in the nucleus and cytoplasm, suggesting the loss of interaction with RNA can be a factor contributing to the escalation of TDP-43 proteinopathy.

## Materials and methods

### Patient samples, exome capture and variant analysis

DNA was available for both the index patient and his father. Post-mortem brain and spinal cord tissue was available from the index patient (donated to the London Neurodegenerative Disease Brain bank with informed consent and under the bank’s ethical approval: 08/MRE09/38+5). Both patients had a diagnosis of definite ALS based on revised El Escorial criteria (Ludolph *et al.*, 2015), and full consent was given for research purposes. DNA was isolated from whole blood and the exome component captured using Roche NimbleGen SeqCap EZ Exome V3 probes. Sequencing was performed on an Illumina Hi-Seq 2000 and 100-bp paired-end reads assembled to the hg19 human reference genome with NovoCraft Novoalign. Variants were called with samtools mpileup, normalized with bcftools norm and annotated using Annovar and additional custom perl scripts. Variants were quality filtered at DP ≥ 10, GQ ≥ 50 and MQ ≥ 50. Variants were excluded if they were present in 1000 Genomes or 670 local control exomes, more than once in UK10K exomes, EVS exomes, or gnomAD genomes, or more than three times in ExAC; frequencies that are comparable to the most common confirmed pathogenic ALS mutations in the ALSoD database. Variants were further excluded if the locus was covered at a depth ≥10× by <10 000 ExAC or gnomAD-genome individuals, or if a focused inspection suggested they were false positive variant calls. For this study, gnomAD-exome is not a suitable resource for filtering rare variants as it contains exomes from several thousand patients with ALS. Assessment of pathogenicity was performed for all non-synonymous variants by 20 different prediction tools available from Annovar. Potential effects on splicing were assessed for variants within 25 bp of a known splice site by ADA, randomForest, Spidex, NetGene2 and GeneSplicer.

Homology modelling of the TDP-43 K181E mutation was performed by Swiss-model, using PDB:4bs2 as a template, and all images were rendered in PyMOL.

### Immunohistochemistry

Sections of 7-μm thickness of human spinal cord tissue samples in 10% formalin-fixed, paraffin-embedded tissue blocks were obtained from the London Neurodegenerative Diseases Brain Bank. The paraffin-embedded tissue blocks were deparaffinized in two changes of xylene and sequential percentage of ethanol solutions for 3 min each (100%, 100%, 95%, 95%, 70%). Antigen retrieval was performed by soaking the slides in 10 mM citrate buffer pH 6.0 and microwaved for 15 min. For diagnosis, paraffin sections were stained for haematoxylin and eosin and sections were immunohistochemically stained with the rabbit polyclonal antibody to phosphorylated TDP-43 (pS409/410-2; 1:1500 Cosmo Bio); mouse monoclonal antibody to phosphorylated tau [clone (AT-8); 1:500; Autogen Bioclear], α-synuclein [clone (42/α-synuclein); 1:500; Novocastra Laboratories Ltd.], and amyloid-β (1:12 000; Chemicon), using the Leica BONDMAX™ (Leica Biosystems). Heat induced epitope retrieval was used for all antibodies except for α-synuclein and amyloid-β. For α-synuclein, and amyloid-β, 80% formic acid pretreatment was used. Nuclei were counterstained with Harris’ alum haematoxylin. For immunofluorescence staining, autofluorescence was blocked by the treatment of Sudan black (0.06 *g* in 20 ml of 70% ethanol) for 10 min. After blocking in normal serum, the sections were stained and imaged as described in the ‘Immunofluorescence’ section.

### Plasmids and antibodies

The GFP-TDP-43 in pEGFP-C1 and HA-TDP-43 in pDEST30 plasmids were generated and used as in previous studies (Nishimura *et al.*, 2010; Scotter *et al.*, 2015; Chen *et al.*, 2016). TDP-43 mutations were introduced using the Q5 Site-Directed Mutagenesis Kit (NEB) with the following mutagenesis primers: K181E forward 5′-CTTCCTAATTCTGAGCAAAGCCAAG-3′ and reverse 5′-GAACCGAAACGAGTCTTAATCCTTC-3′; K181A forward 5′-CTTCCTAATTCTGCGCAAAGCCAAG-3′ and reverse 5′-TTTGCAGTCACACCATCG-3′; D169G forward 5′-CATATGATAGGTGGACGATGG-3′ and reverse 5′-TCGCTGTGACATTACTTTC-3′; K263E forward 5′-CCGAACCTGAGCACAATAGC-3′ and reverse 5′-CATTGGATATATGAACGCTGATTCC-3′. All plasmid sequences were verified by Sanger DNA sequencing.

Primary antibodies used for immunoblotting and immunohistochemistry in this study included: mouse anti-phospho TDP-43 (1:3000, Cosmo Bio), rabbit anti-mouse TDP-43 (0.1 μg/ml, a gift from Prof. Virginia Lee) (Igaz *et al.*, 2011), mouse anti-Actin (1:4000, Sigma), mouse anti-GFP (1:1000, Santa Cruz), rabbit anti-TDP-43 (1:2000, Proteintech), mouse anti-GFAP (1:1000, Abcam) and mouse anti-HA (1:1000, Cell Signaling). Primary antibodies used for immunopurification included rabbit anti-GFP (1:2000, Abcam) and rabbit anti-HA (C29F4) (1:500, Cell Signaling). Primary antibodies used for immunofluorescence included rabbit anti-ubiquitin, K48-specific (1:2000, Millipore), rabbit anti-p62 (1:10 000, Abcam), mouse anti-phospho TDP-43 (1:3000, Cosmo Bio) and rabbit anti-TDP-43 (1:2000, Proteintech).

Secondary antibodies used included DyLight™ 680 goat anti-rabbit IgG (1:10 000, Thermo Scientific), DyLight™ 800 goat anti-mouse IgG (1:10 000, Thermo Scientific), and DyLight™ 488/550/650 anti-rabbit or mouse IgG (1:500, Thermo Scientific).

### Protein construct production and purification for *in vitro* study

The wild-type tandem RRM domains of TDP-43 (RRM1-2, K102-Q269) and the corresponding K181E mutant variant were encoded in a pET-Sumo expression vector containing the kanamycin antibiotic resistance gene. The plasmids were expressed in a Rosetta2(DE3) *Escherichia coli* cell strain as proteins fused with a SUMO solubilization tag carrying a 6×His tag. Cells were grown in Luria-Bertani (LB) medium containing 50 µg/ml kanamycin at 37°C until an optical density of ∼0.7 at 600 nm was reached. Protein expression was induced at 18°C by addition of 0.5 mM IPTG. Cells were collected after overnight growth and resuspended in lysis buffer (10 mM potassium phosphate buffer pH 7.2, 150 mM KCl, 5 mM imidazole, 5% v/v glycerol, 1 mg/ml lysozyme, cOmplete™ EDTA-free Protease Inhibitor tablet by Roche, 1 µg/ml DNase I and 1 µg/ml RNaseA). Cells were lysed by probe sonication and the soluble proteins recovered by centrifugation at 70 000 rcf for 45 min at 4°C. Protein purification included a first nickel affinity chromatography step followed by overnight dialysis in the presence of the Tobacco Etch Virus (TEV) protease at a 1:20 protein:TEV molar ratio to remove the 6×His-SUMO tag. A second nickel-affinity chromatography followed and the flow-through was loaded onto a HiTrap® Heparin column to remove nucleic acids. The protein constructs were finally submitted to size-exclusion chromatography with a HiLoad® 16/60 Superdex 75 prep grade in phosphate buffer pH 7.2. Protein purity was checked by SDS-PAGE.

### Spectroscopic measurements

Circular dichroism (CD) spectra were recorded on a JASCO-1100 spectropolarimeter with a constant N_2_ flush at 4.0 l/min. CD datasets were an average of 15 scans. Far-UV spectrum was recorded at 25°C in phosphate buffer, pH 7.2. Spectra were corrected for buffer signal and expressed as mean residue molar ellipticity θ (deg × cm^2^/dmol).

Uniformly and selectively ^15^N-labelled versions of wild-type RRM1-2 and the K181E mutant were produced by standard methods (Marley *et al.*, 2001). 2D nuclear magnetic resonance (NMR) ^15^N-HSQC (heteronuclear single quantum coherence) spectra were recorded at 700 MHz frequency at 25°C on Varian spectrometers.

### Aggregation kinetics assay

Protein aggregation was monitored by following the increment in emission fluorescence of the aggregate-specific dye ProteoStat® on a FLUOstar Omega plate reader. Proteins were diluted in phosphate buffer pH 7.2 to a concentration of 10 μM, with and without equal amount of the AUG-RNA (5′-GUGUGAAUGAAU-3′). The plate was sealed with an optic seal and the assay plates were incubated at 37°C under, shaking for 2 s (≈200 rpm) before each read (every 15 min). The experiments were performed at least in triplicate and the results referred to the blank, normalized and reported as percentage average.

### Cell culture and DNA transfection

HEK293T and SH-SY5Y cells were cultured using Dulbecco’s modified Eagle medium (DMEM) and DMEM/F12 (Invitrogen) supplemented with 10% foetal bovine serum (Invitrogen), and maintained at 37°C, 5% CO^2^. Cells were plated a day before transfection and media was refreshed before plasmid DNA transfection using Lipofectamine™ 2000 (Invitrogen). Cells were left for 48 h after transfection to be harvested for analysis unless otherwise stated.

### RNA extraction and RT-PCR

RNA was extracted using RNeasy® Mini Kit (Qiagen). cDNA was generated using 1 μg of RNA, oligo d(T) primer and SuperScript™ III First-Strand Synthesis Kit (Invitrogen). Fifty nanograms of cDNA was used for RT-PCR with forward primers labelled with IRDye 700 at the 5′ end. Primers used in this study included: *POLDIP3* forward 5′-TGCTCTGAAGCTCACCAAAA-3′ and reverse 5′-GGAACGGAAGCTATACCATCAT-3′ (Tollervey *et al.*, 2011); EGFP forward 5′-CTG AAGTTCATCTGCACCAC-3′ and reverse 5′-GGTCTTGTAGTTGCCGTCG-3′; *GAPDH* forward 5′-CCTGACCTGCCGTCTAGAAA-3′ and reverse 5′-ATCCTGGTGCTCAGTGTAGCC-3′. RT-PCR products were analysed by 2% agarose gel. Images were taken by the Odyssey or GelDoc imaging system and quantified by ImageJ (http://imagej.nih.gov/ij/).

### Solubility fractionation

The fractionation for protein solubility was performed using a protocol described by Winton *et al.* (2008) with some minor modifications (Chen *et al.*, 2016). Cells were harvested in RIPA buffer (150 mM NaCl, 1% NP-40, 0.5% sodium deoxycholate, 0.1% SDS, 50 mM Tris pH 8.0 and protease and phosphatase inhibitors), sonicated and centrifuged at 12 000*g* for 20 min at 4°C. After centrifugation, the supernatant was collected as the RIPA solubility fraction. The pellet, after being washed once with RIPA buffer, was then suspended in 20% of the original lysis volume with urea buffer (7 M Urea, 2 M thiourea, 4% CHAPS and 30 mM Tris pH 8.5) and collected as the insoluble, detergent-resistant fraction.

### Immunopurification

Cells were harvested in IP buffer (50 mM Tris pH 7.4, 150 mM NaCl, 1% Triton™ X-100 with protease and phosphatase inhibitor). After a short centrifuge (14 000 rpm for 30 s at 4°C), the supernatant was collected and pre-cleaned with Dynabead® protein G (Invitrogen) 2 h at 4°C. The pre-cleaned lysate was then incubated with immunopurification antibody and fresh Dynabead® protein G (Invitrogen) overnight at 4°C. The Dynabead® protein G-antibody-protein complex was purified using magnetic separation and washed with IP buffer before elution in loading buffer.

### Western blotting and densitometry analysis

Protein quantification and western blotting were performed as described before (Nishimura *et al.*, 2010; Chen *et al.*, 2016). Five micrograms of cell lysate from the RIPA fraction and the equivalent liquid volume from the urea fraction were loaded. Western blot quantification were performed used the image analysis software, ImageJ (http://imagej.nih.gov/ij/). Integrated band intensities were normalized to that of loading control or the RIPA fraction.

### Immunofluorescence

Cells for immunofluorescent analyses were fixed in 4% paraformaldehyde (VWR) for 20 min and washed with phosphate-buffered saline (PBS) three times for 5 min. Cells were permeabilized by incubation in PBS containing 0.5% Triton™ X-100 (Sigma) for 15 min at room temperature, followed by blocking in PBS containing 1% donkey serum for 1 h at room temperature. Cells were incubated with primary antibody diluted in blocking solution overnight at 4°C. After washing in PBS, cells were subsequently incubated with fluorescent secondary antibodies diluted in blocking solution for 1 h at room temperature. DAPI (Sigma) was then used to stain for nuclei before being mounted on coverslips using FluroSave (Calbiochem).

### RNA electrophoretic mobility-shift assay

Using the LightShift Chemiluminescent RNA EMSA Kit (electrophoretic mobility-shift assay, EMSA) (Thermo Fisher), 5 μg of total cell lysate in RIPA containing RNase inhibitor (NEB) was mixed with 2 μg of tRNA, 1 μM of unlabelled (GU)×6 RNA and 2.5 nM of biotin-labelled (GU)×6 RNA (CGUGUGUGUGUGUGGU) (Bhardwaj *et al.*, 2013) and was incubated for 30 min at room temperature. The binding reactions were run on a 6% non-denaturing polyacrylamide gel, followed by semi-dry transfer (3 mA/cm^2^, 30 min) to Hybond-N+ nylon membrane (GE Healthcare) and UV cross linking (120 mJ/cm^2^). Biotin-labelled RNA was detected by chemiluminescence with stabilized streptavidin-horseradish peroxidase conjugate provided by the RNA EMSA kit.

### Fluorescence recovery after photobleaching

HEK293T cells (10^6^) per well were plated on the 15μ-Slide 8 well chamber slide (Ibidi) the day before transfection. Cells were transfected with GFP-TDP-43 for 48 h and Hoechst staining (1 mg/ml, Thermo Fisher) was applied 20 min before imaging.

Fluorescence recovery after photobleaching (FRAP) was performed using a Nikon A1R laser scanning confocal microscope fitted with an environmental chamber maintained at 37°C. Confocal images of 512 × 512 pixels were acquired using a ×60 oil immersion objective. Five frames were acquired before bleaching (10% laser power, 4 frames/s of 10 loops) a 3 × 3 μm^2^ region of interest. Fluorescence recovery was followed for 1 min after bleaching. Data analysis was performed using Nikon Elements software as per manufacturer’s instruction. The EGFP fluorescence intensity at each time point was measured for the bleached region of interest as well as for an unbleached reference region of interest of the same size to correct for acquisition bleaching. FRAP recovery for the bleached region of interest was calculated by first subtracting the reference at each time point and then normalized to the pre-bleached fluorescence intensity.

### Data availability

The data supporting the findings of this study are available from the corresponding author on request. 

## Results

### Identification of novel RRM-adjacent *TARDBP* mutation in a familial ALS/FTD kindred

**Figure 1:**
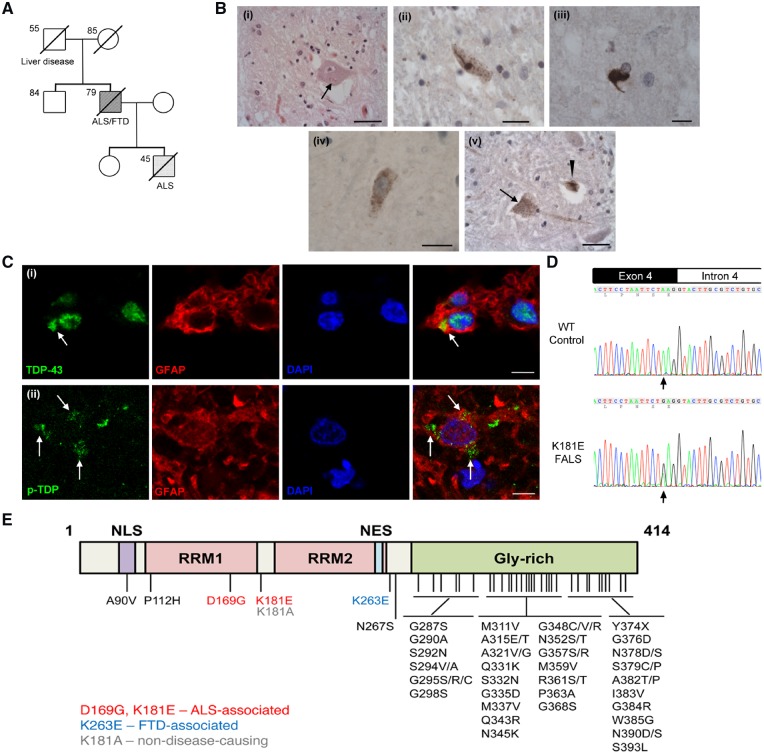
**Identification of the K181E mutation in a family of ALS/FTD.** (**A**) The pedigree of the ALS/FTD kindred carrying the K181E mutation is shown. Affected individuals are indicated by shaded symbols, deceased individuals with a slash, and numbers indicate current age or age at death. (**B**) Brain and spinal cord pathology of index patient. [**B**(**i**)] Bunina bodies (arrow) observed in a slightly atrophic anterior horn neuron from the spinal cord. [**B**(**ii**)] Granular/skein-like pTDP-43-positive cytoplasmic inclusion in an atrophic anterior horn neuron from the spinal cord. [**B**(**iii**)] A pTDP-43-positive glial cytoplasmic inclusion within the spinal cord. [**B**(**iv**)] A motor neuron from the motor cortex containing granular cytoplasmic pTDP-43-positive inclusions. [**B**(**v**)] The XIIth nerve nucleus of the medulla showing skein-like (arrowhead) and granular (black arrow) cytoplasmic pTDP-43-positive neuronal inclusions. Scale bar = 50 µm in **i**, **ii**, **iv**, **v**; 20 µm in **iii**. (**C**) Cytoplasmic TDP-43 [**C**(**i**), arrow] and p-TDP-43-positive inclusions [**C**(**ii**), arrows] observed in GFAP-positive cells. Images were taken in the area of anterior horn from the spinal cord. Scale bar = 5 µm. (**D**) Sanger sequencing chromatogram demonstrating a wild-type control and a heterozygous single base substitution (c.541A>G) predicted to substitute lysine for glutamic acid (p.Lys181Glu, K181E) in the index patient and his father. (**E**) Schematic overview of TDP-43 protein domain structure and disease-associated mutations.

A 38-year-old male patient presented with predominantly lower motor neuron signs that progressed over 48 months to a flaccid tetraparesis, but he was still able to speak and had normal cognitive function. His father developed lower motor neuron signs in his upper limbs at the age of 76 and 6 months later developed major personality and behavioural changes consistent with a clinical diagnosis of FTD that eventually led to profound cognitive deficits. Brain MRI was consistent with a diagnosis of FTD. The index patient survived 80 months from symptom onset and his father survived 36 months (Fig. 1A). The brain and spinal cord tissues from the index patient were available for further neuropathological examination

The formalin fixed left half of the brain weighed 782 g. There was some slight cerebral swelling noted but no cerebral atrophy. No other macroscopical abnormality was seen in the brain. The spinal cord did show some evidence of thinning of anterior nerve roots. Histologically the spinal cord showed loss of anterior horn neurons and some surviving neurons contained Bunina bodies [Fig. 1B(i)]. Immunohistochemistry revealed phosphorylated (p)TDP-43-positive skein-like inclusions in the neurons [Fig. 1B(ii)], and numerous pTDP-43-positive cytoplasmic inclusions in glial cells [Fig. 1B(iii)] . There was loss of myelin in the lateral and anterior corticospinal tracts of the cord, indicating upper motor neuron loss. Indeed within the motor cortex there was loss of Betz cells, and many surviving Betz cells contained pTDP-43-positive neuronal [Fig. 1B(iv)] cytoplasmic inclusions. There were pTDP-43-positive neuronal [Fig. 1B(v)] and glial cytoplasmic inclusions in the XIIth nerve nucleus of the medulla, and also the midbrain, basal ganglia and amygdala but not the hippocampus or neocortex. The glial cells containing cytoplasmic TDP-43 inclusions were GFAP-positive (Fig. 1C) but MBP-or CD68-negative (data not shown), showing that cytoplasmic TDP-43 accumulation was present in astrocytes but not seen in oligodendrocytes or microglia. There was no tau, amyloid-β or α-synuclein pathology present.

NimbleGen exome capture and Illumina sequencing were carried out on whole blood DNA for both the father and son, resulting in 134 million and 160 million 100-nucleotide paired-end reads, respectively, which when assembled to the human reference genome, covered 91.76% of the coding bases in Refseq to a depth ≥10× in both samples. A relaxed filtering strategy identified 29 candidate variants present in both affected family members (Supplementary Table 1), 11 of which were not present in the 1000 Gnomes, UK10K, EVS/ESP, ExAC, or gnomAD variant databases, nor in 670 local controls. One of these variants was of paramount interest as it represented a novel missense change in the RNA-binding domain of TDP-43 (*TARDBP*: NM_007375:c.541A>G:p.Lys181Glu) (K181E), predicted to be damaging by 15 of 20 prediction tools used to assess pathogenicity. The heterozygous mutation of TDP-43 K181E was validated with Sanger DNA sequencing (Fig. 1D) and is predicted to substitute a positively-charged lysine (K) residue adjacent to the first RNA-binding domain with a negatively-charged glutamic acid (E). The K181E mutation lies adjacent to the RRM1 whereas most ALS-associated mutations cluster at the ‘prion-like’ domain (Fig. 1E)

### The structure of the tandem RRM domains is not altered by the K181E mutation

We speculated on a possible destabilizing effect of the K181E mutation on the structure of TDP-43. Because of the known difficulties in working with full-length TDP-43 *in vitro* (Johnson *et al.*, 2009), we produced a construct containing the two tandem RRM domains of TDP-43, here called RRM1-2, as heterologous protein expressed in *E. coli*, both as wild-type and as a K181E mutant to investigate the mutation effect.

**Figure 2:**
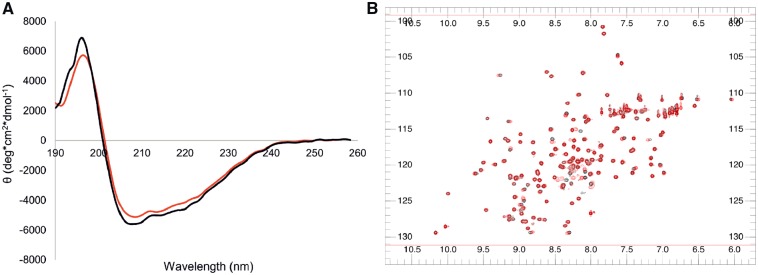
**Structural comparison of the wild-type and K181E tandem RRM domains.** (**A**) CD spectra acquired at room temperature, indicating almost complete overlap in their secondary structure. (**B**) Superposition of heteronuclear single quantum coherence (HSQC) experiments showing that the two variants have nearly identical NMR spectra. Black = wild-type RRM1-2; red = K181E RRM1-2.

CD spectroscopy was used to compare the overall secondary structure of the two variants (Fig. 2A). Both spectra displayed the signature typical of folded α-β proteins and overlapped both in shape and signal intensity, indicating that the secondary structure is not altered by the K181E mutation. We also investigated the potential local effect of the mutation by NMR HSQC spectroscopy (Fig. 2B). The 2D spectra resulted highly similar, both in terms of chemical shifts and relative resonance intensities, indicating that mutation of K181 to E does not affect the chemical environment of the protein.

These results clearly exclude that the K181E mutation could alter the structure of the tandem RRM motifs and led us to believe that the effect on the native conformation of full-length TDP-43 is not structural.

### K181E decreases TDP-43 protein solubility and enhances hyperphosphorylation

**Figure 3:**
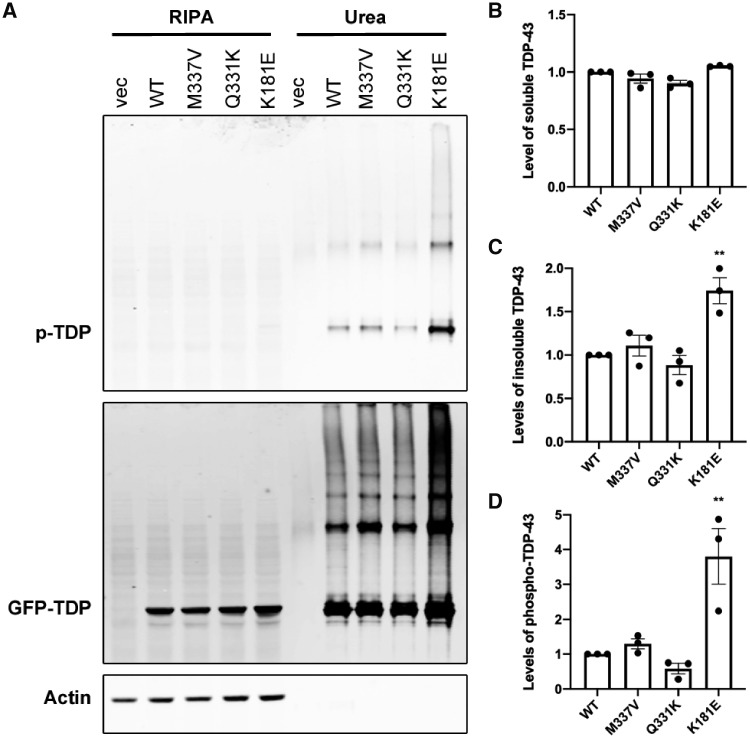
**The K181E mutation increases TDP-43 detergent-resistance and phosphorylation.** (**A**) HEK293T cells expressing GFP-TDP-43 constructs for 48 h, followed by fractionation. Phospho-TDP-43 specific antibody is used to visualize phospho-TDP-43 and TDP-43 antibody for total overexpressed GFP-TDP-43. Levels of GFP-TDP-43 (**B** and **C**) and phospho-TDP-43 (**D**) from three independent transfections were quantified, normalized to actin and shown in relation to GFP-WT TDP-43. Mean and standard error of the mean (SEM) are shown for the soluble (**B**), and insoluble (**C** and **D**) fractions. The data were analysed by one-way ANOVA followed by Bonferroni post-test. Significant increases in detergent-resistance and protein phosphorylation is found with K181E-TDP-43 (*P* < 0.01 for both). WT = wild-type.

In ALS and FTD, pathological TDP-43 protein accumulates in affected neurons, forming hyperphosphorylated and detergent-resistant aggregates. In an earlier study, we observed elevated levels of insoluble and hyperphosphorylated TDP-43 following the overexpression of both wild-type and ALS-associated C-terminal TDP-43 mutants (Chen *et al.*, 2016). To investigate whether the novel RRM-domain K181E mutation is pathological and causes TDP-43 aggregation, we used the same cellular model transiently overexpressing EGFP-tagged full length TDP-43 in HEK293T cells. Levels of C-terminal mutant M337V and Q331K protein in the urea-soluble fraction was similar to wild-type protein but levels of insolubility and phosphorylation of the K181E mutant protein were increased 2- to 4-fold, respectively (Fig. 3). Similar results were also found when the same EGFP-TDP-43 constructs were expressed in the neuroblastoma cell line, SH-SY5Y (Supplementary Fig. 1), validating the profound effect of the K181E mutation in enhancing features of TDP-43 proteinopathy. Although the K181E mutation lies well outside the C-terminal domain and does not have a significant impact on the structure of the RRM1-2 area (Fig. 2), the increased levels of hyperphosphorylated and insoluble TDP-43 protein are consistent with it being pathological.

### K181E disrupts TDP-43 binding to target RNA and promotes nuclear aggregation

**Figure 4:**
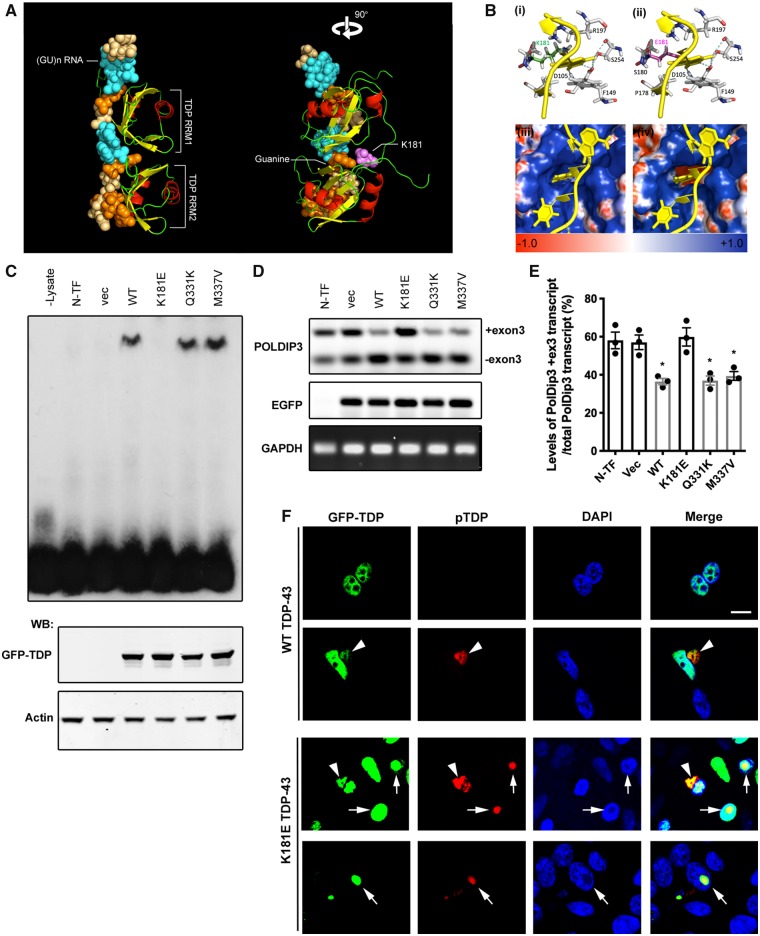
**The K181E mutation disrupts the interaction between TDP-43 protein and RNA interaction, which prevents splicing of target RNA and increases TDP-43 aggregating.** (**A**) Crystal structure of TDP-43 N-terminal fragment (pdb:4bs2) containing two RRM domains (red helices, yellow beta sheets) interacting with a GU-rich RNA strand (blue/orange spheres), showing TDP-43 lysine 181 (purple spheres) to be in close contact with a guanine nucleotide. (**B**) Close-up of the guanine-binding pocket formed by D105, F149, K181, R197 and S254 (**i**) is not altered structurally altered by the E181 mutation (**ii**); however, the positively-charged cavity (**iii**) gains a strongly negative electrostatic charge in the presence of the ALS-linked mutation (**iv**). (**C**) RNA-EMSA using total cell lysate from 48 h GFP-TDP-43 transfected HEK293T cells and biotin-labelled (GU)×6 RNA. Western blots (WB) of GFP-TDP-43 actin are used to demonstrate uniform expression of GFP-TDP-43 and protein loading; *n* = 3. (**D**) Endogenous *POLDIP3* mRNA splicing assay using RNA extracted from 48 h GFP-TDP-43 transfected HEK293T cells. RT-PCR for *POLDIP3*, *EGFP* and *GAPDH* was carried out to show the TDP-43-mediated mRNA splicing activity, transfection efficiency and loading. (**E**) The levels of *POLDIP3* long and short transcripts from three independent studies were quantified. Means and SEMs are shown. The data were analysed by one-way ANOVA followed by Bonferroni post-test. Wild-type, Q331K- and M337V-TDP-43 significantly alter the splicing profile of *POLDIP3* (*P* < 0.05) whereas K181E-TDP-43 does not have any significant impact, compared to untransfected or GFP-only control. (**F**) Phospho-TDP-43 staining (red) in HEK293T cells transfected for 48 h with GFP wild-type or K181E-TDP-43. Scale bar = 10 μm.

The majority of ALS-associated TDP-43 mutations have so far been found to occur within the low complexity ‘prion-like’ C-terminal domain (amino acids 274 to 414, Fig. 1E), which is involved in interactions with other proteins (Buratti *et al.*, 2005; D'Ambrogio *et al.*, 2009; March *et al.*, 2016). Although the mechanism(s) of C-terminal mutant protein toxicity are not clear, they have a longer half-life than wild-type TDP-43 and are more prone to form cytoplasmic aggregates (Barmada *et al.*, 2010; Ling *et al.*, 2010). The K181E mutation, however, is adjacent to the RRM1 domain, which plays a dominant role in DNA and RNA binding (Buratti and Baralle, 2001; Kuo *et al.*, 2009; Che *et al.*, 2015). The crystal structure of a peptide containing both TDP-43 RRM domains bound to a GU-rich-RNA strand has been resolved (PDB:4bs2) and shows that the lysine residue at 181 lies within one wall of a pocket-like cavity into which a guanine nucleotide is bound (Fig. 4A, B and Supplementary Video 1; Lukavsky *et al.*, 2013). As the lysine residue is positively charged, it will have a greater affinity for negatively-charged DNA or RNA nucleotides than the negatively-charged mutant glutamic acid (E), which is predicted to create an electrostatic repulsion between the protein and the nucleotide (Fig. 4B) that could reduce the RNA-binding capacity of TDP-43. We therefore investigated the interaction between the TDP-43 protein and a known RNA target, (GU)×6 (Bhardwaj *et al.*, 2013) using an electromobility shift assay (EMSA). Wild-type GFP-TDP-43 showed highly efficient binding to (GU)×6 RNA, as did Q331K and M337V TDP-43 (Fig. 4C); however, this interaction was completely abolished by the K181E mutation (Fig. 4C). This loss of RNA-binding of K181E TDP-43 was also shown by its inability to regulate the splicing of its target RNAs, such as *POLDIP3* (Fig. 4D and E). Wild-type, Q331K and M337V all enhanced the exclusion of *POLDIP3* exon 3 but K181E failed to splice out this exon (Fig. 4D and E). Interestingly, in addition to the occasional cytosolic hyperphosphorylated aggregates seen in wild-type TDP-43 overexpression, K181E TDP-43 frequently formed nuclear aggregates that were also hyperphosphorylated (Fig. 4F), which implies that RNA binding in the nucleus may prevent aberrant TDP-43 aggregation.

The impact of K181E mutation on RNA interaction observed in RNA-EMSA was validated in an *in vitro* study where we used the RRM1-2 wild-type and K181E mutant constructs and determined the dissociation constant (K_d_) of the interaction with AUG RNA by means of biolayer interferometry. We found that wild-type RRM1-2 binds the UG-rich AUG RNA with a K_d_ of 3.2 ± 0.9 nM, while the K181E mutant displayed a K_d_ of 2.5 ± 0.4 µM (Supplementary Fig. 2).

We can thus conclude that, while not having any structural relevance, the mutation leads to severe impairment of function and prevents the interaction between TDP-43 protein and its target RNA, which is crucial for effective splicing and trafficking. Our data have also shown that the K181E-TDP-43 is unable to bind and process RNA and is highly prone to aggregation.

### Disruption of RNA binding enhances TDP-43 phosphorylation and aggregation

**Figure 5:**
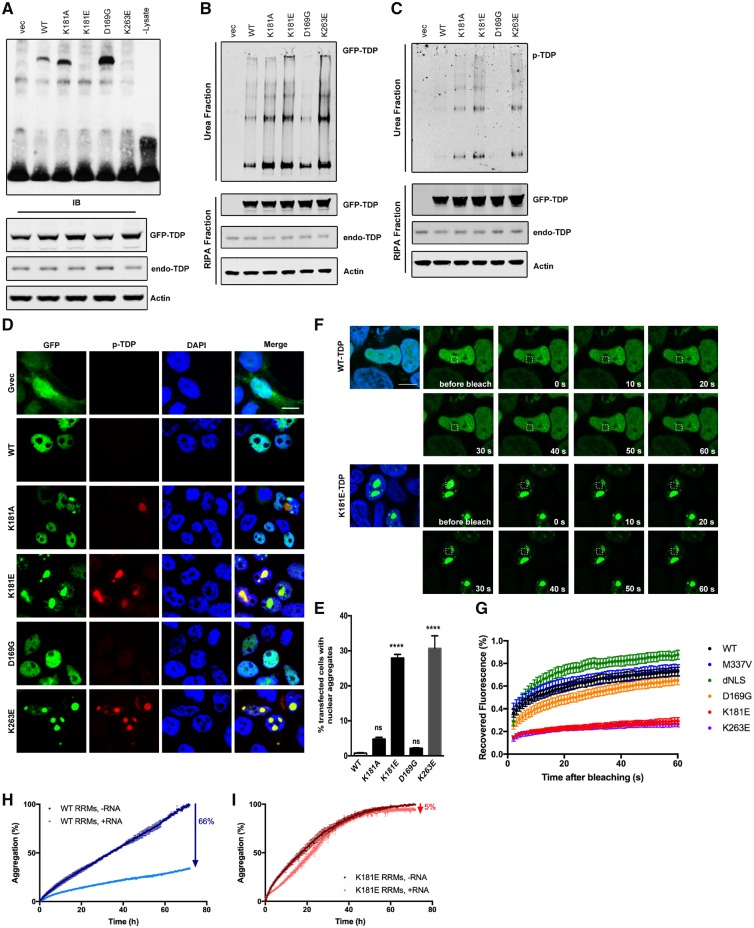
**Disruption of RNA binding enhances TDP-43 protein aggregation and phosphorylation.** (**A**) RNA EMSA using total cell lysate from 48 h GFP-TDP-43 transfected HEK293T cells and biotin-labelled (GU)×6 RNA. Western blots of GFP-TDP-43 and actin demonstrate the equal expression of GFP-TDP-43 constructs and protein loading. (**B** and **C**) HEK293T cells express GFP-TDP-43 constructs for 48 h followed by fractionation. GFP or phospho-TDP-43-specific antibody was used to visualize total GFP-TDP-43 (**B**) or phospho-TDP-43 (**C**). (**D** and **E**) Phospho-TDP-43 staining (red) in HEK293T cells transfected for 48 h with GFP-TDP-43. Scale bar = 10 μm. Cell counting was carried out manually under fluorescence microscope. At least 200 GFP-positive cells were counted for each condition, per transfection, using an unbiased protocol as described previously (Lee *et al.*, 2013). One-way ANOVA and Bonferroni post-test shows that there are significantly more nuclear TDP-43-containing cells in both K181E- and K263E-TDP-43-expressing conditions (*P* < 0.0001). (**F** and **G**) FRAP was carried out after 48 h of GFP-TDP-43 transfection in HEK293T cells. A 3 × 3 μm^2^ area (indicated with white box) was bleached with fluorescence and recovery recorded for 1 min after bleaching. Hoechst 33342 staining (blue) was used to visualize the nucleus. Scale bar = 10 μm. Five cells were bleached and recorded for each condition, per transfection. Means and SEMs of three transfections are plotted in **G**. (**H** and **I**) Fluorescence-associate aggregation kinetics in the absence and presence of RNA. The percentage of aggregation reduction of the wild-type-RRM1-2 (**H**) or K181E-RRM1-2 (**I**) protein constructs due to the presence of RNA is indicated by an arrow at a 1:1 protein:RNA molar ratio. All experiments were repeated at least three times.

To dissect the relationship between RNA binding and TDP-43 proteinopathy further, we extended our study to other missense variants that occur in or around the RRM domains, including K181A, D169G and K263E. The K181A variant is an artificial construct generated to test the affinity of RNA for the RRM1 of TDP-43, which was shown to be unaffected (Fig. 5A; Lukavsky *et al.*, 2013). The D169G mutation was identified in one sporadic ALS case (Kabashi *et al.*, 2008) and shown not to have any impact on RNA binding in *in vitro* studies despite its location at the RRM1 domain (Fig. 5A; Austin *et al.*, 2014; Kuo *et al.*, 2014), nor functional disruption to full length TDP-43 (McDonald *et al.*, 2011; Vanden Broeck *et al.*, 2015). We were able to confirm that neither K181A nor D169G variants disrupt RNA binding (Fig. 5A). Alanine has a neutral charge and is therefore less likely to repel nucleotides at position 181, while the substitution of aspartic acid (D) to glycine (G) at positon 169 is distant from the RNA-binding interface and unlikely to cause direct steric or electrostatic disruption (Supplementary Fig. 3). K263E was identified in one patient diagnosed with sporadic FTD (Kovacs *et al.*, 2009) and in cellular studies this mutation was shown to increase TDP-43 stability and enhance its ubiquitination (Austin *et al.*, 2014; Hans *et al.*, 2014). Here we show that, like K181E, the K263E mutation also disrupts the capacity of TDP-43 to bind RNA (Fig. 5A). Interestingly, K263 in the RRM2 domain shares structural and functional homology with the K181 residue in the RRM1 domain, in that it contributes to forming a positively-charged groove into which a guanine nucleotide is shown to fit. The negatively-charged glutamic acid (E) residue in this position in the RRM2 domain is also predicted to repel negatively-charged nucleotides and inhibit RNA binding.

The K181E and K263E mutants also had much higher levels of phosphorylation and reduced solubility compared to wild-type- and the D169G-TDP-43 mutation on western blots (Fig. 5B and C) and were more likely to form phosphorylated intra-nuclear aggregates (30% of transfected cells, Fig. 5D and E) compared to wild-type-, D169G- and K181A-TDP-43, which have a diffuse distribution in the nucleus (Fig. 5D and E). Although K181A did not disrupt RNA binding in our *in vitro* assay (Fig. 5A), it did show a modest increase in detergent-resistant phosphorylated intra-nuclear aggregates (Fig. 5C and D). FRAP analysis of the nuclear TDP-43 aggregates demonstrated that they were highly immobile compared to diffuse nuclear wild-type, D169G or M337V TDP-43, or the nuclear localization signal (NLS) deleted cytoplasmic (dNLS) TDP-43 (Fig. 5F, G and Supplementary Videos 2 and 3).

Recently, we showed that UG-rich RNA can significantly reduce the aggregation of TDP-43 RNA-recognition regions (Zacco *et al.*, 2019). We used the aggregation detection fluorophore ProteoStat, which allows us to monitor aggregate formation as a function of time. We normalized the data as referred to the value reached at end of the assay (3 days). In the absence of UG-rich RNA, wild-type RRM1-2 exhibited time-dependent self-assembly (Fig. 5H). When RNA was added in a 1:1 ratio, the percentage of aggregates recorded for the wild-type after 3 days was only 34% the value detected in the absence of RNA, indicating a RNA-dependent aggregation inhibition of RRM1-2 aggregation of 66% (Fig. 5H). When the same level of RNA was added to the K181E mutant aggregate formation was reduced only by 5% (Fig. 5I), suggesting that the weak interaction with this RNA translate into a weak inhibitory effect on the aggregation behaviour.

The amino acids surrounding K181 are the most conserved region in TDP-43, in particular the 109 amino acids from the C-terminal half of RRM1 (Phe152) to the end of RRM2 (Ala260), which are completely devoid of any nonsense or missense variants in the ExAC database (Supplementary Fig. 4). This high level of conservation indicates that this region plays a crucial role in TDP-43 functionality and that there has been a strong selection pressure against changes to the RRM domains. Specific mutations such as K181E and K263E in this highly conserved region disrupt RNA binding and promote the accumulation of detergent resistant, hyperphosphorylated, immobile nuclear TDP-43 aggregates conferring likely pathogenicity. It is also interesting to note that the capacity to interact with RNA may play an important role in maintaining TDP-43 solubility and functionality.

### K181E-TDP-43 interacts and recruits wild-type endogenous TDP-43 protein to nuclear aggregates

**Figure 6:**
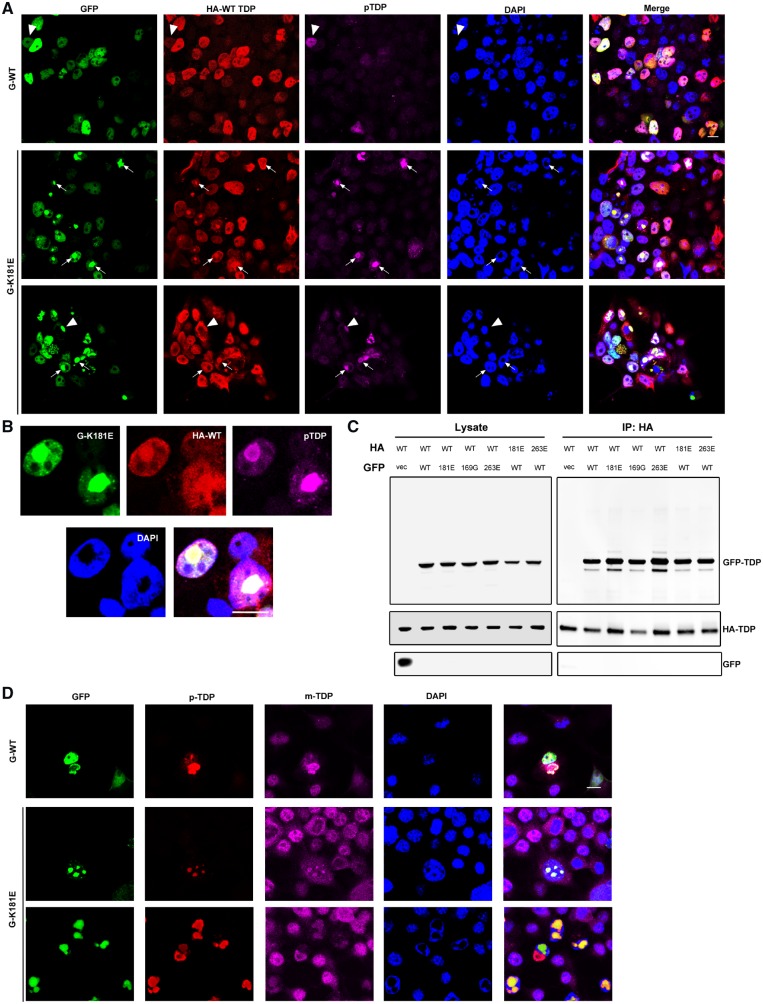
**K181E mutant TDP-43 binds to wild-type-TDP-43 and sequesters it in phosphorylated intranuclear and cytoplasmic inclusions.** (**A** and **B**) HEK293T cells co-expressing GFP WT- or K181E-TDP-43 and HA WT-TDP-43 for 48 h were fixed and stained with HA (red) and phospho-TDP-43 (magenta). Cytoplasmic hyperphosphorylated TDP-43 aggregates (arrowhead) were seen in both conditions; nuclear hyperphosphorylated aggregates containing both wild-type (WT) and K181E-TDP are indicated by arrows. (**C**) Co-immunopurification of GFP-WT or mutant TDP-43 and HA wild-type-TDP-43. HA WT-TDP-43 was pulled down by the HA antibody and GFP-TDP-43 detected with the TDP-43 antibody. (**D**) Interaction between GFP-K181E TDP-43 and endogenous wild-type TDP-43 in the mouse neuronal cell line, N2a. After 48 h transfection with GFP-TDP-43, cells were fixed and stained by phospho-TDP-43-specific antibody (red) and the mouse TDP-43-specific antibody (magenta). Scale bar = 10 μm. All experiments were repeated at least three times.

TDP-43 is an aggregation-prone protein that forms oligomers under physiological conditions and detergent-resistant aggregates in disease and overexpression cellular and transgenic models (Neumann *et al.*, 2006; Mitchell *et al.*, 2015; Chen *et al.*, 2016; Afroz *et al.*, 2017). To investigate the interaction between mutant and wild-type proteins, we co-expressed wild-type and K181E HA- and GFP-tagged constructs (Fig. 6A). Overexpression of haemagglutinin (HA)- and GFP-tagged wild-type-TDP-43 generated occasional hyperphosphorylated TDP-43 cytoplasmic aggregates that consisted of both HA- and GFP-wild-type TDP-43 proteins. Co-aggregation was far more abundant in wild-type- and K181E-TDP-43 co-expressing cells predominantly within the nucleus (Fig. 6A and B). The interaction between wild-type- and mutant-TDP-43 proteins was confirmed as co-immunoprecipitation of HA-tagged wild-type and mutant TDP-43 consistently pulled down the GFP-tagged wild-type TDP-43 protein, which was most abundant with HA-K181E and K263E bait (Fig. 6C). Thus aggregation of mutant or wild-type protein in the cytoplasm or nucleus is able to recruit endogenous wild-type protein (Fig. 6D) and could thereby interfere with RNA processing.

In summary, we have shown that although K181E and K263E ALS and FTD-associated mutants of TDP-43 do not directly bind RNA, they are able to disrupt RNA processing by sequestering endogenous wild-type TDP-43 protein within hyperphosphorylated aggregates, which could have significant functional consequences.

### Disruption of cytoplasmic TDP-43 RNA binding aggravates TDP-43 proteinopathy

**Figure 7:**
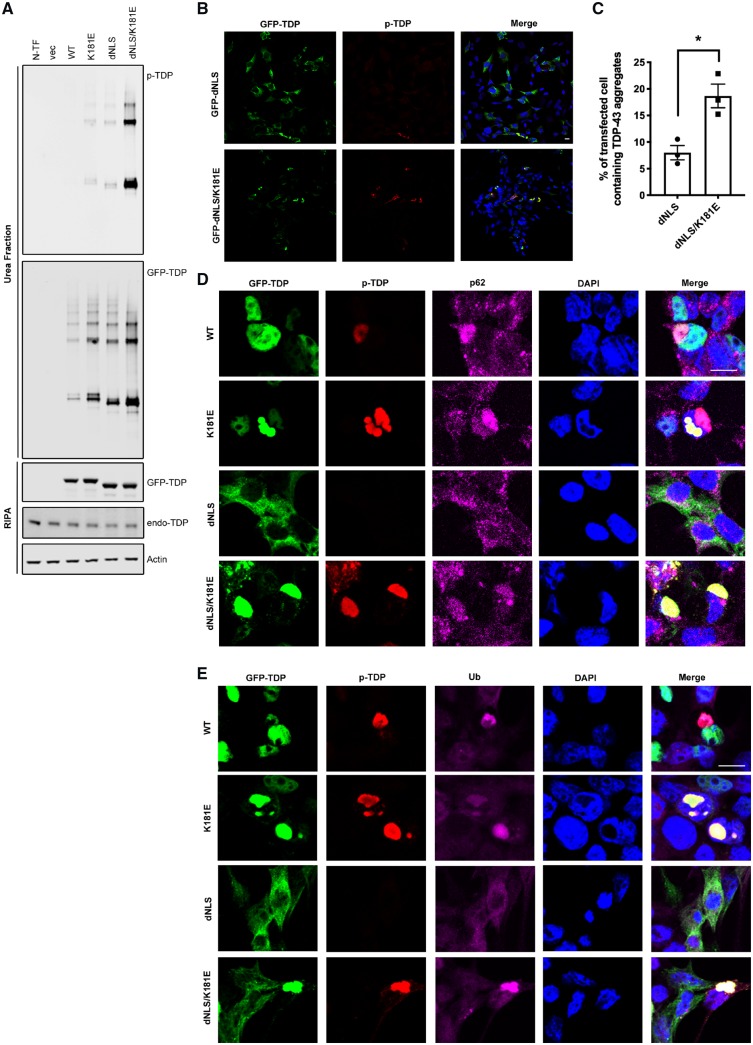
**Disruption of cytoplasmic TDP-43 RNA binding aggravates TDP-43 proteinopathy.** (**A**) HEK293T cells expressing GFP-TDP-43 constructs for 48 h followed by fractionation. GFP and phospho-TDP-43-specific antibodies were used to visualize total GFP-TDP-43 and phospho-TDP-43. Endogenous TDP-43 proteins are shown by TDP-43 antibody, and actin is shown as a loading control. (**B**–**E**) HEK293T cells expressing GFP-TDP-43 for 48 h were fixed and stained with phospho-TDP-43 (red), p62 (magenta; **D**) and ubiquitin (magenta; **E**). DAPI (blue) is used to visualize the nucleus. Scale bar = 10 μm. The percentage of GFP-dNLS and GFP-dNLS K181E TDP-43 aggregation were quantified via cell counting as described before (Lee *et al.*, 2013). Means and SEMs are shown in **C.** All experiments were repeated at least three times.

TDP-43 is predominantly a nuclear protein; however, it is known to shuttle between the nucleus and cytoplasm, playing a crucial role in regulating RNA transport, metabolism and local cytoplasmic translation (Liu-Yesucevitz *et al.*, 2011; Fallini *et al.*, 2012; Yasuda and Mili, 2016). To investigate the influence of RNA binding on the behaviour of cytoplasmic TDP-43, we introduced the K181E mutation into a TDP-43 construct lacking the nuclear localizing signal (GFP-dNLS TDP-43), which is preferentially retained in the cytoplasm. Interestingly, the K181E mutation substantially increased levels of hyperphosphorylated TDP-43 detergent resistant aggregates (Fig. 7A–C and Supplementary Fig. 5A), which were localized to the cytoplasm in both HEK293T and SH-SY5Y cells (Fig. 7B, C and Supplementary Fig. 5B). Protein aggregates associated with neurodegenerative disorders are often decorated with proteins that facilitate protein degradation such as ubiquitin and p62 (Arai *et al.*, 2003; Neumann *et al.*, 2006). Although nuclear and cytoplasmic aggregates of wild-type and mutant TDP-43 were labelled with ubiquitin, p62 stains more intensively with cytoplasmic aggregates and weaker with nuclear aggregates associated with the K181E mutation (Fig. 7D and E).

The formation of hyperphosphorylated TDP-43 cytoplasmic and, less commonly, nuclear aggregates, are a hallmark feature of ALS and FTD, but the factors that contribute to their development are not fully understood. We have confirmed that some disease-associated mutations adjacent to RRM1 and RRM2 disrupt RNA binding and form nuclear and cytoplasmic aggregates. This may impair RNA processing through the sequestration of wild-type protein, acting in a dominant-negative fashion. Furthermore, the binding to its target RNA plays an important role in maintaining the solubility of TDP-43 protein. Disruption of RNA binding promotes TDP-43 to aggregate in the cytoplasm as well as in the nucleus.

## Discussion

Mutations in *TARDBP* linked to ALS and FTD and are associated with phosphorylated cytoplasmic TDP-43 aggregates in degenerating neurons (Sreedharan *et al.*, 2008; Kovacs *et al.*, 2009; Synofzik *et al.*, 2014). Using exome sequencing, we detected a novel K181E *TARDBP* mutation in a father and son with classical ALS/FTD and ALS, respectively, and we excluded mutations in all other known ALS and FTD genes. More than 90% of all TDP-43 mutations are located in the low-complexity C terminal domain and the pathogenicity of this variant, which is situated adjacent to the dominant DNA/RNA-binding domain RRM1, was unknown. Our 3D modelling showed that K181E and K263E, but not D169G, were predicted to reduce the positive charge within the nucleotide binding site. Here we have confirmed that K181E and K263E, but not D169G, disrupted RNA binding to these mutant proteins and abolished splicing of its endogenous RNA target *POLDIP3*. The loss of RNA binding by these two mutants was associated with a dramatic increase in TDP-43 protein phosphorylation, insolubility and aggregation, predominantly within the nucleus and to a lesser extent in the cytoplasm. We have demonstrated that wild-type TDP-43 is recruited to mutant aggregates that may exert a dominant-negative effect on RNA processing in the nucleus and cytoplasm.

### TDP-43-mediated RNA regulation in health and disease

As a DNA/RNA-binding protein, TDP-43 is known to physically bind to mRNAs and miRNAs to regulate their splicing, stability, transport and translation (Ayala *et al.*, 2005; Buratti *et al.*, 2010; Polymenidou *et al.*, 2011; Tollervey *et al.*, 2011; Humphrey *et al.*, 2017). In addition to its role in normal RNA processing, TDP-43 has recently been shown to serve as a chaperone for toxic RNA, such as the UGGAA repeat expansion associated with spinocerebellar ataxia type 31 (SCA31) (Niimi *et al.*, 2013; Ishiguro *et al.*, 2017). Functional disruption of TDP-43 RRM domains abolishes this interaction and results in the accumulation of (UGGAA)n RNA foci and repeat-associated pentapeptide, which leads to neurodegeneration (Ishiguro *et al.*, 2017). On the other hand, because of its role in RNA regulation, changes in TDP-43 protein levels impact on a vast number of its target RNAs, leading to alterations in the levels and splicing patterns of the cellular RNA profile (Polymenidou *et al.*, 2011; Zhang *et al.*, 2012). Knockdown of TDP-43 in cells or animals impacts widely on the levels and splicing pattern of a vast number of RNA transcripts (Polymenidou *et al.*, 2011; Ling *et al.*, 2015). Whereas the investigations into altered RNA profiles of ALS patients verify some of the changes observed in the knockdown study, the variance between human studies are simply too big to draw a consistent conclusion (Polymenidou *et al.*, 2011; Shiga *et al.*, 2012; Brohawn *et al.*, 2016; D'Erchia *et al.*, 2017).

A loss-of-function hypothesis has long been speculated as a mechanism underlying TDP-43-associated disease pathogenesis. Systematic knockout of TDP-43 in mice results in embryonic lethality (Kraemer *et al.*, 2010; Sephton *et al.*, 2010; Wu *et al.*, 2010) indicating that TDP-43 plays a crucial irreplaceable role during early embryo development. On the other hand, targeted depletion of TDP-43 in adult mouse motor neurons induces ALS-like progressive motor dysfunction and muscle atrophy (Wu *et al.*, 2012; Iguchi *et al.*, 2013), with similar results reported in *Drosophila* (Feiguin *et al.*, 2009; Fiesel *et al.*, 2010; Lin *et al.*, 2011; Diaper *et al.*, 2013) and *Caenorhabditis elegans* (Vaccaro *et al.*, 2012; Zhang *et al.*, 2012). Collectively, these studies indicate that TDP-43 plays an import role in maintaining the function and wellbeing of motor neurons. Interestingly, knockdown of endogenous TDP-43 shows the same impact on *POLDIP3* splicing as overexpression of wild-type TDP-43 alone (Figs 4C and 5D) (Fiesel *et al.*, 2010; Shiga *et al.*, 2012; Suzuki *et al.*, 2015), which indicates that TDP-43 needs to be maintained at a critical level, lower and higher protein levels are both likely to result in function disturbance and eventual cytotoxicity.

RRM1 has a very high affinity for RNA and plays a major role in mediating TDP-43/RNA interactions whereas RRM2 has a lower affinity for RNA and has a predominantly regulatory function (Buratti and Baralle, 2001; Lukavsky *et al.*, 2013; Kuo *et al.*, 2014). Functional mutation or complete deletion of the RRM2 domain has a limited impact on RNA binding (Buratti and Baralle, 2001); however, we have shown that both the K181E and K263E mutations completely abolish the binding of TDP-43 to its target RNA, and implying that the RRM2 does play an important role in RNA processing. The loss of RNA binding generated abundant insoluble hyperphosphorylated TDP-43 aggregates which are the hallmark of TDP-43 proteinopathy in FTD/ALS supports the case that these mutations are pathogenic. Interestingly, unlike the C-terminal mutations clustered in the glycine-rich domain that do not affect RNA binding, both K181E and K263E have been identified in families with FTD with or without ALS phenotypes. Whereas TDP-43 proteinopathy is commonly seen in tau-negative FTD, the mutations in TDP-43 found in FTD are still rare. Our study showed that in addition to not binding to RNA, both K181E and K26E mutations increase the insolubility and hyperphosphorylation of TDP-43. It is not yet clear whether this would subsequently trigger a distinct pathogenesis mechanism that is more likely to affect cognitive function. Further *in vivo* studies are needed to investigate the link between the loss of TDP-43 RNA binding and the cognitive dysfunction.

The presence of TDP-43 cytoplasmic inclusions is the hallmark of ALS and FTD but nuclear clearing is not always present, particularly in spinal cord motor neurons in ALS (Neumann *et al.*, 2007; van der Zee *et al.*, 2009; Weihl, 2011). Many cellular and animal models of TDP-43 overexpression show neurotoxicity in the absence of nuclear clearance (Barmada *et al.*, 2010; Mitchell *et al.*, 2015; Liu *et al.*, 2017) thus the aggregates themselves may be directly toxic to multiple cellular processes involving; intracellular transport, endoplasmic reticulum, mitochondria and proteostasis as a gain-of-function mechanism. They could also lead to the sequestration of soluble TDP-43 in the periphery, causing loss of normal TDP-43 protein transport and translation as a dominant-negative effect. TDP-43 RRM mutant proteins may be unable to process RNA and therefore auto-regulate its own translation, they do form aggregates and can sequester wild-type protein so both gain- and loss-of-function effects may contribute to neurodegeneration.

### RNA chaperone

Interestingly, not only can the TDP-43 protein act as a chaperone to protect neurons from toxic RNA (Ishiguro *et al.*, 2017), RNA may in turn play a role in keeping TDP-43 soluble and preventing it from aggregating. Target single-stranded DNA or RNA has been shown to enhance TDP-43 protein solubility *in vitro* (Huang *et al.*, 2013; Sun *et al.*, 2014) and the addition of an RNA-binding domain to the aggregation-prone *E. coli* lysol tRNA synthetase increased its protein folding and solubility in the presence of RNA (Choi *et al.*, 2008). On the other hand, TDP-43 containing the artificial mutations F147/149L, which functionally disrupt the RRM1, forms nuclear aggregates in cellular models and remains insoluble following the addition of RNA (Ayala *et al.*, 2008; Huang *et al.*, 2013). These results are consistent with our observation that RNA-free TDP-43 misfolds, becomes hyperphosphorylated and aggregates in the nucleus and cytoplasm indicates that the interaction with RNA plays an important role in maintaining TDP-43 protein solubility, and could be explored as a mean of therapeutic intervention for ALS.

In conclusion, we have identified a novel mutation adjacent to the RRM1 domain in a father and son with ALS and ALS/FTD. RRM domains show marked evolutionary conservation and variation largely absent from human genome databases. We have shown that two TDP-43 mutations, adjacent to either RRM domain, will abolish RNA binding and promote the formation of hyperphosphorylated, insoluble TDP-43 aggregates that are the hallmark of FTD and ALS, providing functional evidence that these mutations are likely to be pathogenic. Mutant TDP-43 aggregates may be directly toxic but they can also sequester wild-type TDP-43 protein and the loss of RNA processing function, including the autoregulation of *TARDBP* (TDP-43) mRNA, would be predicted to affect a multitude of important cellular pathways that could lead to neurodegeneration. As more wild-type TDP-43 becomes trapped within aggregates, less soluble TDP-43 is available to the cell. Eventually the RNA binding deficient mutant TDP-43 can deprive the cell of its functional RNA processing counterpart, resulting in a dominant-negative loss-of-function. Furthermore, our studies imply that RNA binding to TDP-43 can inhibit its aggregation, which may open new avenues for therapeutic intervention.

## Supplementary Material

awz313_Supplementary_Materials

## Abbreviations

ALS =: amyotrophic lateral sclerosis
FTD =: frontotemporal dementia
