# Supplementary material for: RRM adjacent TARDBP mutations disrupt RNA binding and enhance TDP-43 proteinopathy
Source: Brain. 2019 Oct 11;142(12):3753–70. doi: 10.1093/brain/awz313 (PMC6885686; doi:10.1093/brain/awz313)
Supplement: awz313_Supplementary_Materials [file awz313_supplementary_materials.zip › awz313-suppl_data/Supplementary blot.pdf]

Supplementary blot: RRM adjacent TARDBP mutations disrupt RNA binding and enhance TDP-43 proteinopathy

Han-Jou Chen[1,2 *], Simon D. Topp[1], Ho Sang Hui[1], Elsa Zacco[1], Malvika Katarya[1], Conor McLoughlin[1], Andrew King[3], Bradley N. Smith[1], Claire Troakes[3], Annalisa Pastore[1] , Christopher E. Shaw[1,4 *]

Figure 3A

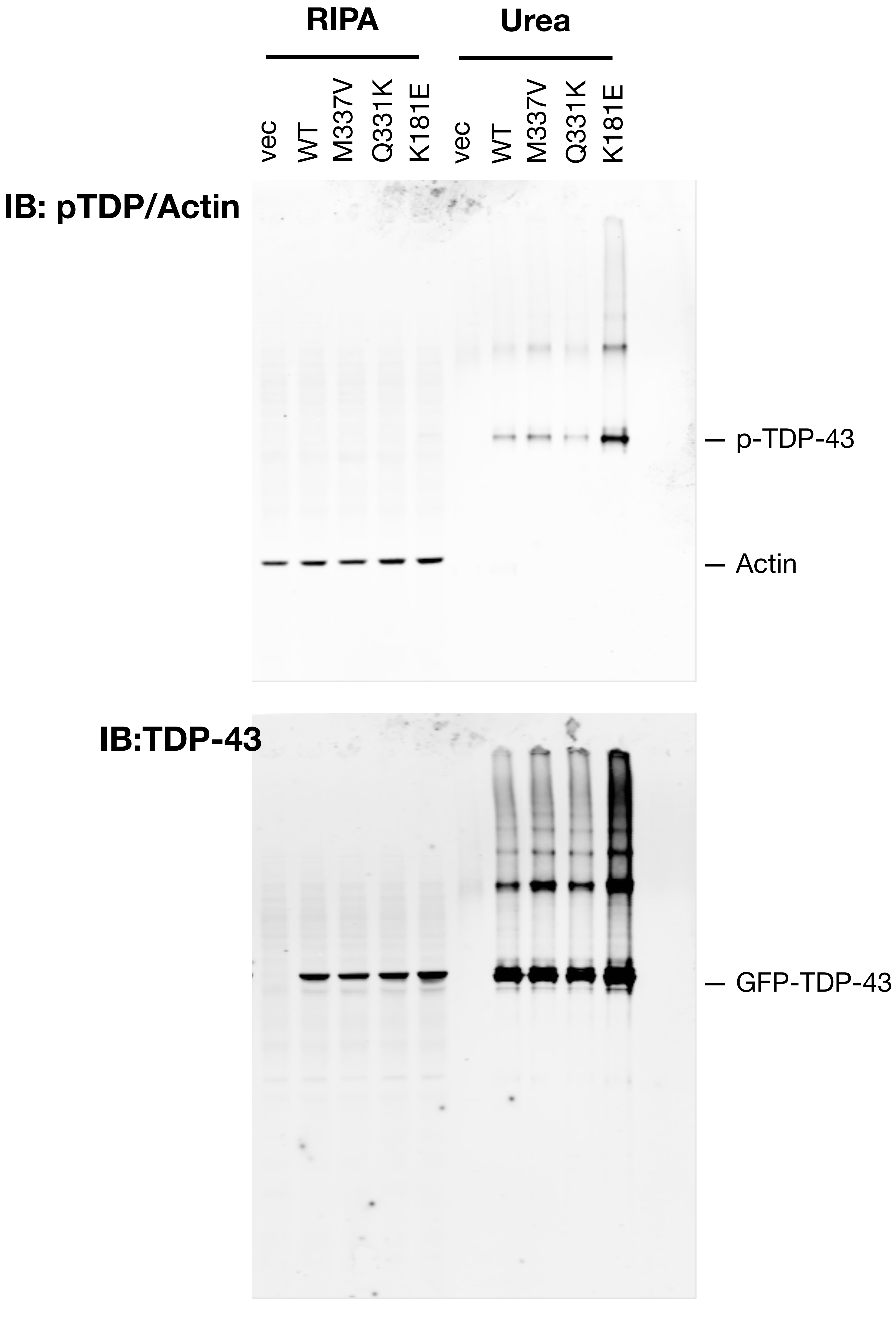
RIPA
Urea
vec
WT
M337V
Q331K
K181E
vec
WT
M337V
Q331K
K181E
IB: pTDP/Actin
— p-TDP-43
— Actin
IB:TDP-43
— GFP-TDP-43

Figure 4C

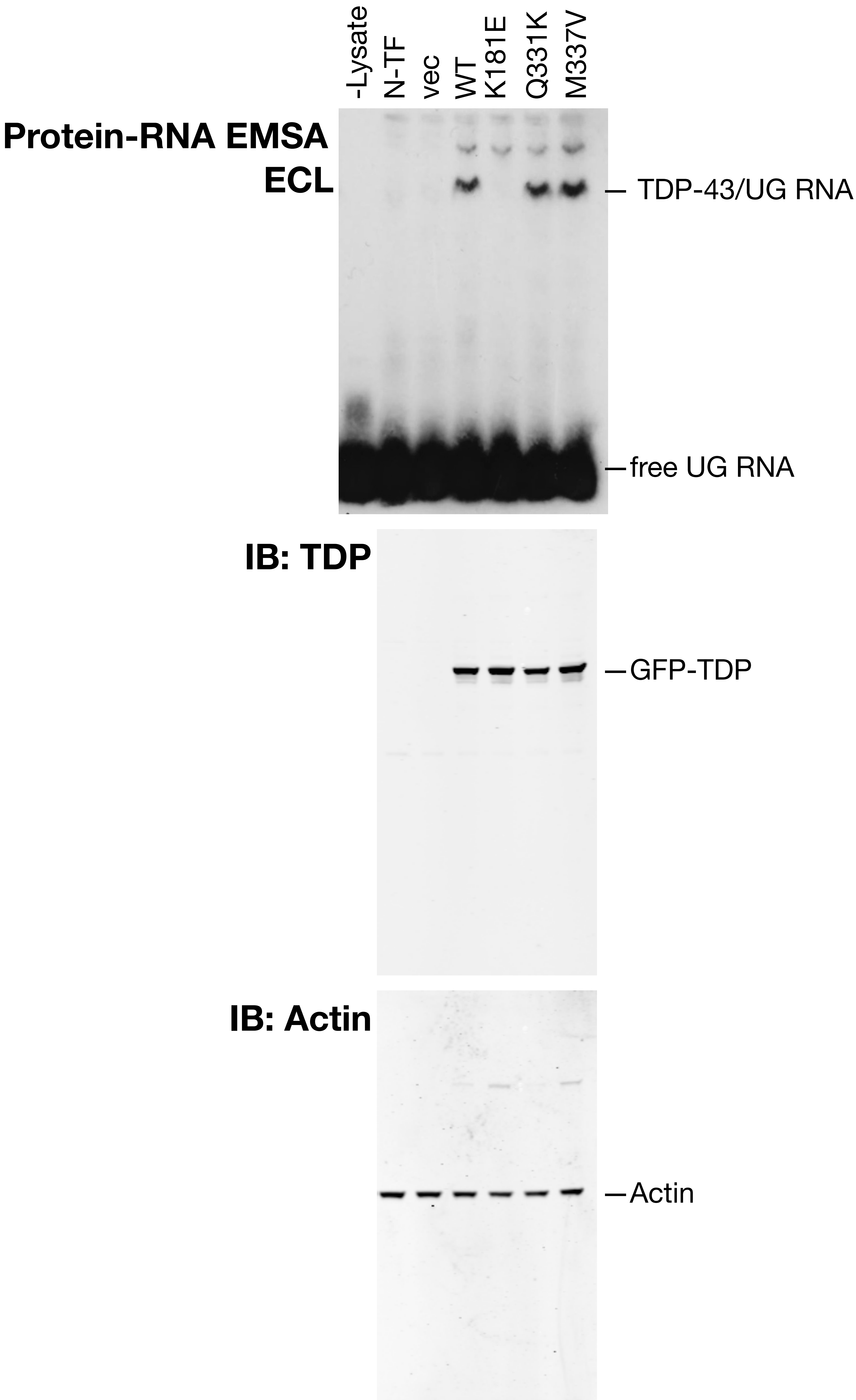

-Lysate
N-TF
vec
WT
K181E
Q331K
M337V
Protein-RNA EMSA
ECL
TDP-43/UG RNA
free UG RNA
IB: TDP
GFP-TDP
IB: Actin
Actin

Figure 4D

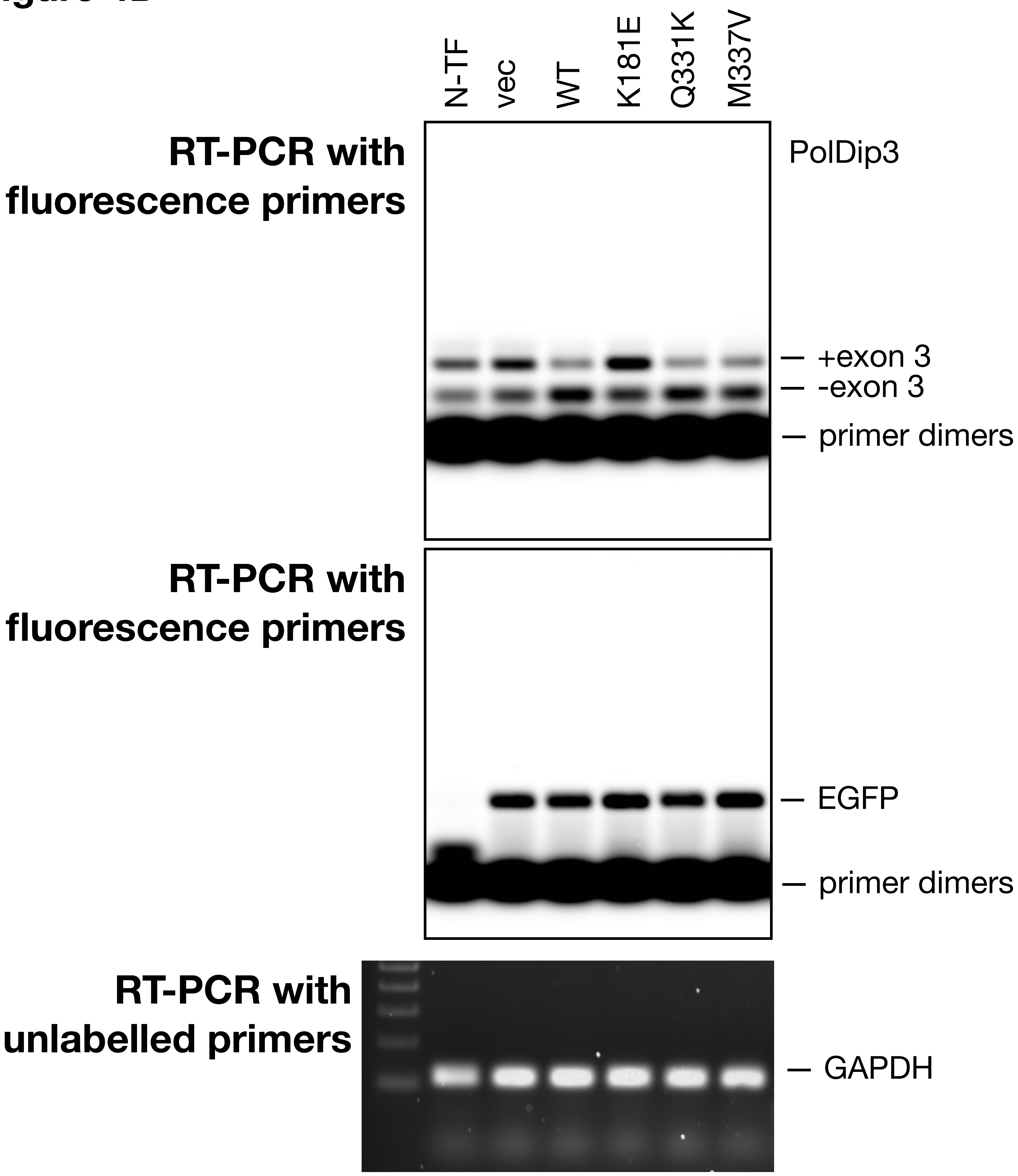
N-TF
vec
WT
K181E
Q331K
M337V
RT-PCR with fluorescence primers
PolDip3
+exon 3
-exon 3
primer dimers
RT-PCR with fluorescence primers
EGFP
primer dimers
RT-PCR with unlabelled primers
GAPDH

Figure 5A

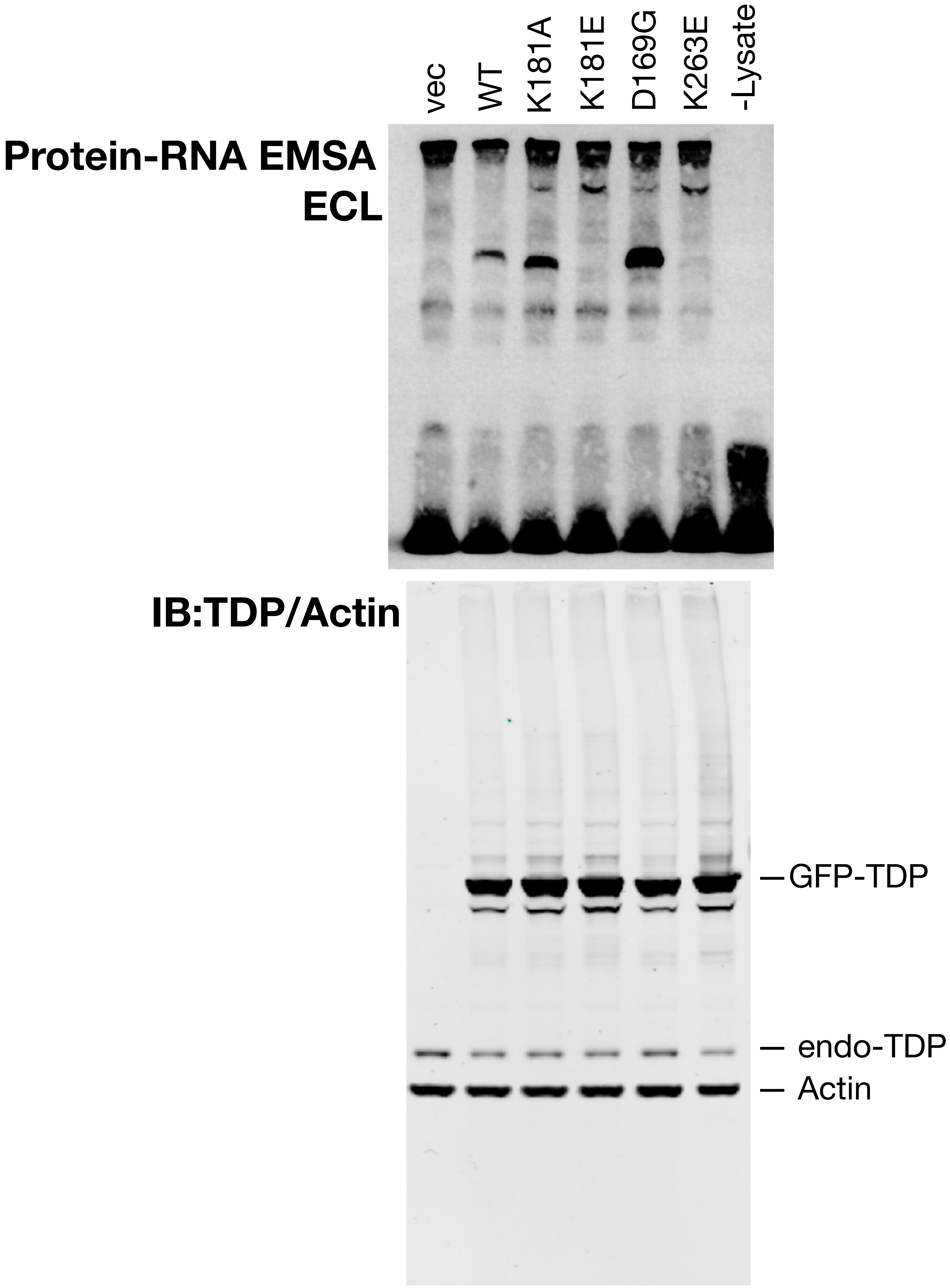

vec
WT
K181A
K181E
D169G
K263E
-Lysate
Protein-RNA EMSA
ECL
IB:TDP/Actin
GFP-TDP
endo-TDP
Actin

Figure 5B-C

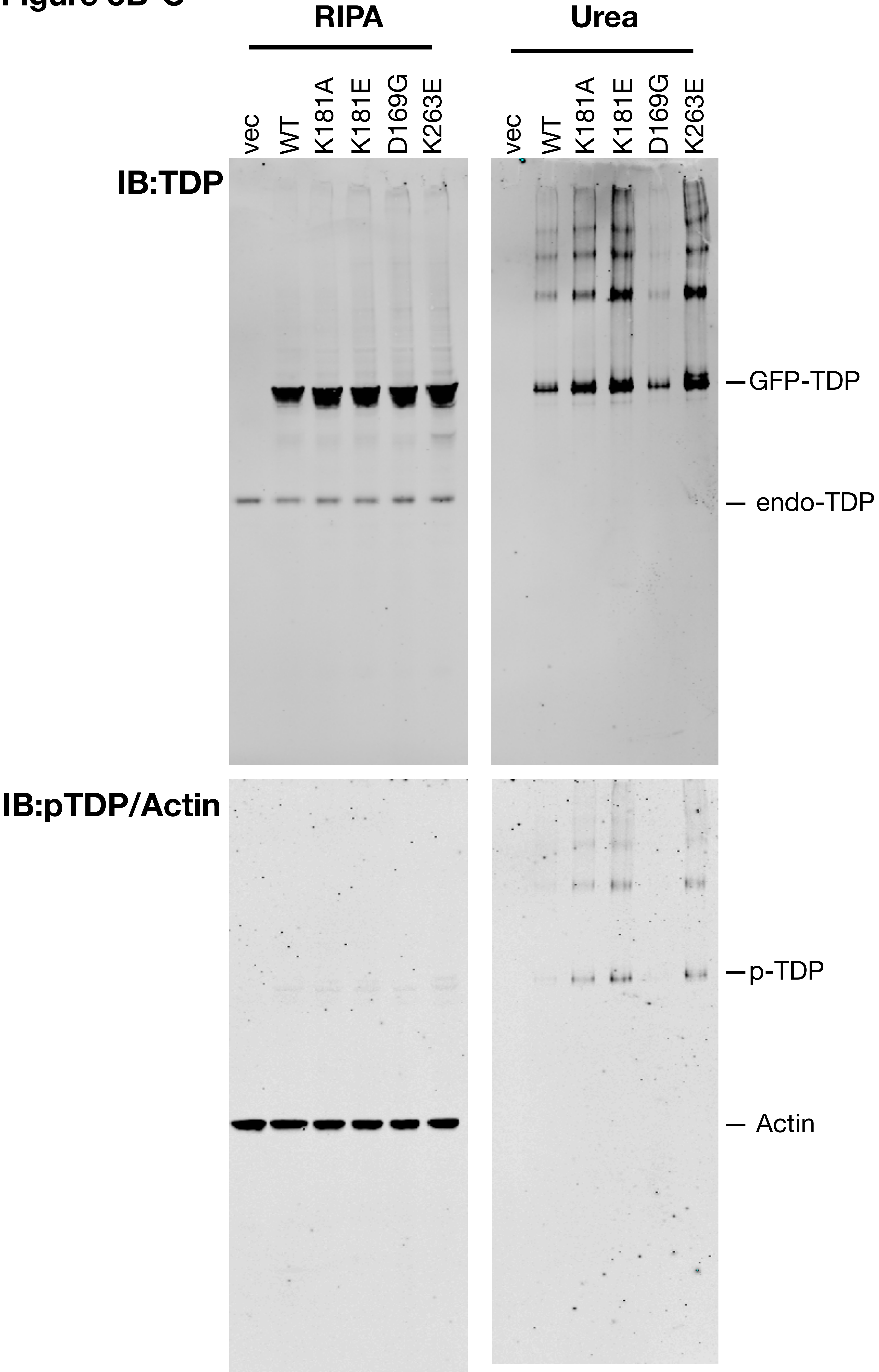
RIPA
Urea
vec
WT
K181A
K181E
D169G
K263E
vec
WT
K181A
K181E
D169G
K263E
IB:TDP
GFP-TDP
endo-TDP
IB:pTDP/Actin
p-TDP
Actin

Figure 6C

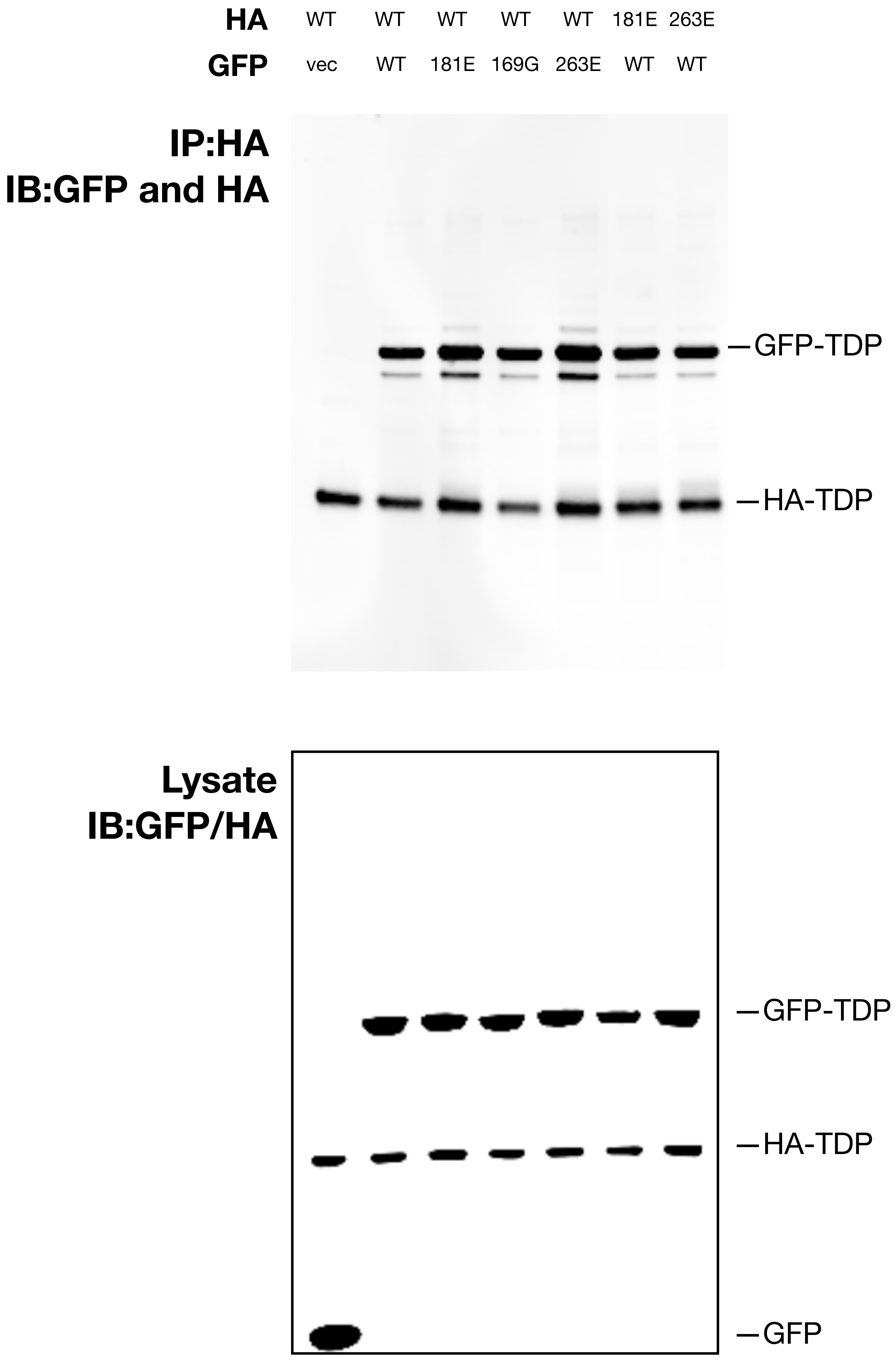
HA WT WT WT WT WT 181E 263E
GFP vec WT 181E 169G 263E WT WT
IP:HA
IB:GFP and HA
GFP-TDP
HA-TDP
Lysate
IB:GFP/HA
GFP-TDP
HA-TDP
GFP

Figure 7A

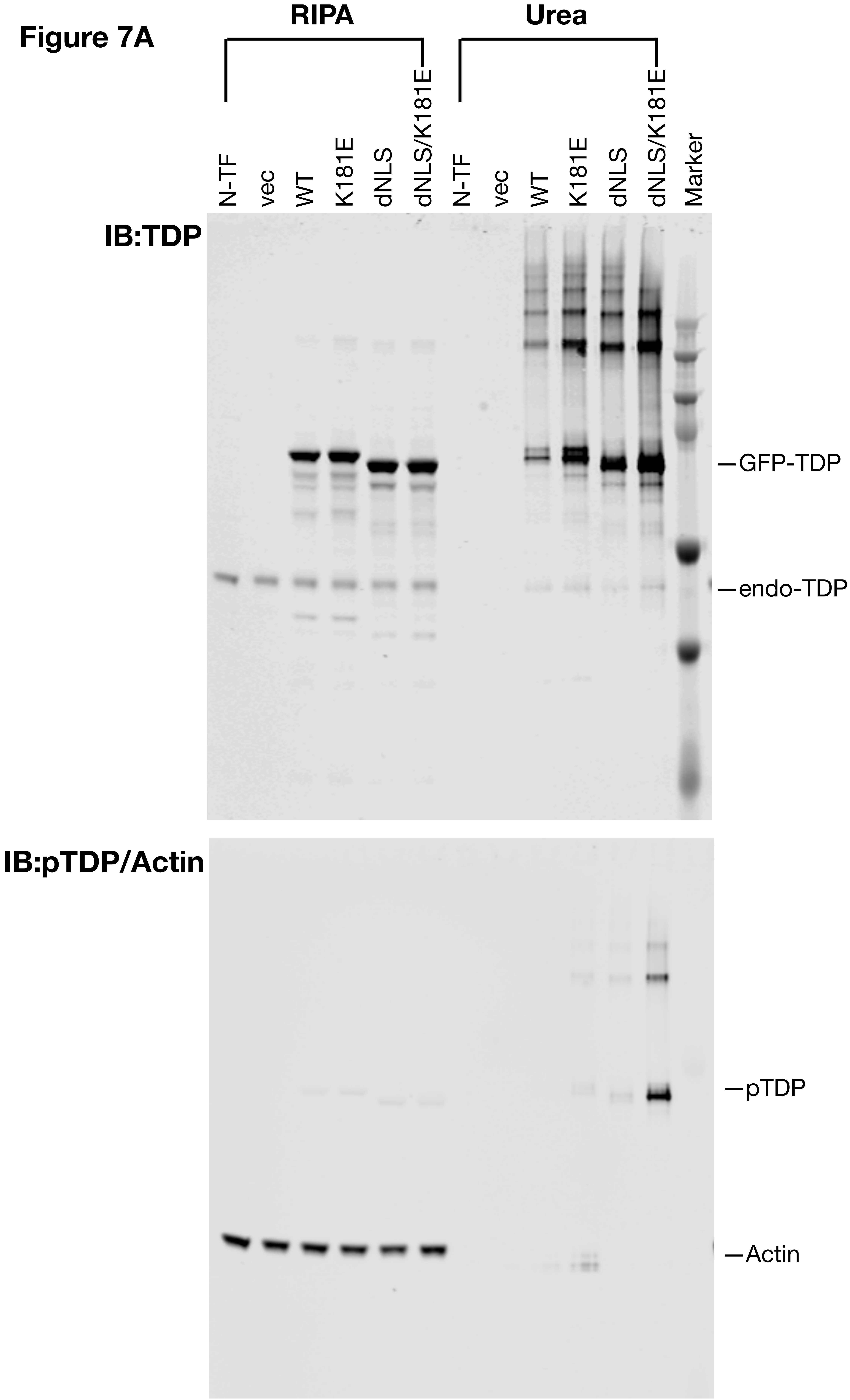
RIPA
Urea
N-TF
vec
WT
K181E
dNLS
dNLS/K181E
N-TF
vec
WT
K181E
dNLS
dNLS/K181E
Marker
IB:TDP
GFP-TDP
endo-TDP
IB:pTDP/Actin
pTDP
Actin
