# Supplementary material for: RRM adjacent TARDBP mutations disrupt RNA binding and enhance TDP-43 proteinopathy
Source: Brain. 2019 Oct 11;142(12):3753–70. doi: 10.1093/brain/awz313 (PMC6885686; doi:10.1093/brain/awz313)
Supplement: awz313_Supplementary_Materials [file awz313_supplementary_materials.zip › awz313-suppl_data/Supplementary materials.pdf]

Supplementary materials

RRM adjacent TARDBP mutations disrupt RNA binding and enhance TDP-43 proteinopathy

Han-Jou Chen[1,2 *], Simon D. Topp[1], Ho Sang Hui[1], Elsa Zacco[1], Malvika Katarya[1], Conor McLoughlin[1], Andrew King[3], Bradley N. Smith[1], Claire Troakes[3], Annalisa Pastore[1, 4,] , Christopher E. Shaw[1,5 *]

Supplementary Figure 1

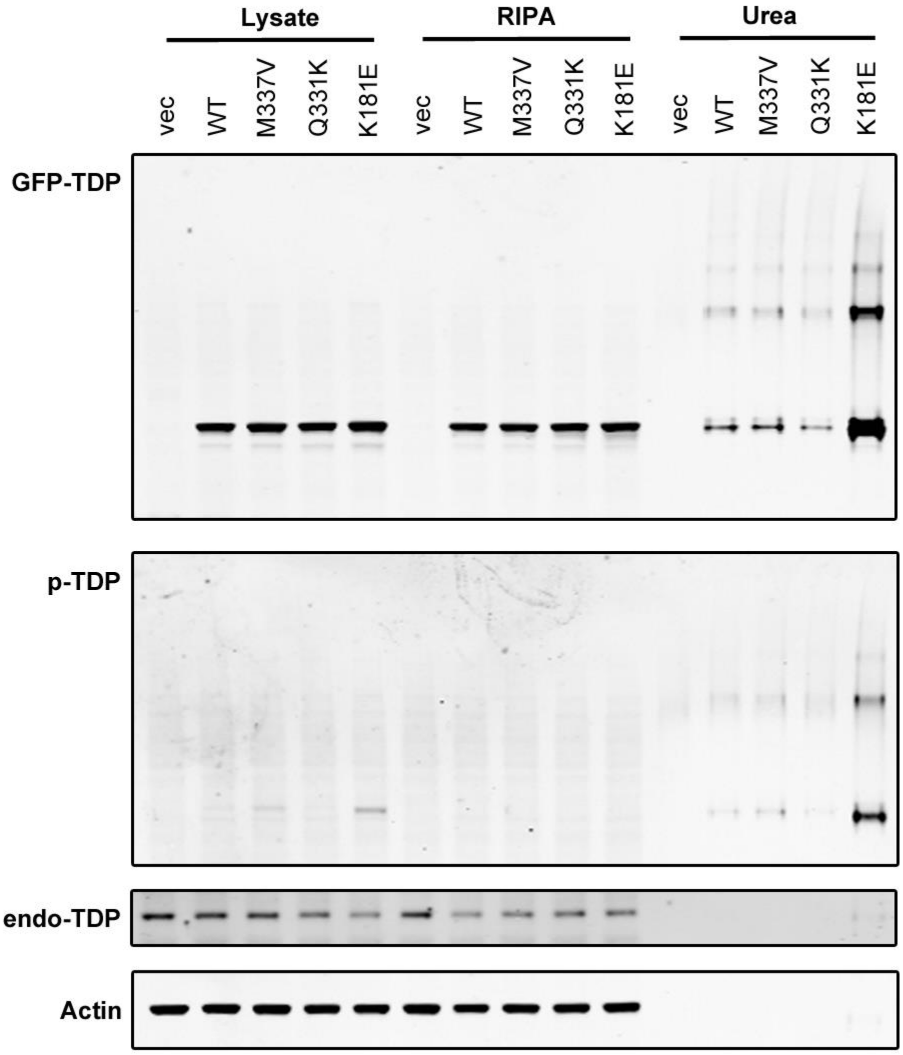

The K181E mutation aggravates TDP-43 protein phosphorylation and detergent resistance in SH-SY5Y cells. The human neuroblastoma cell line, SH-SY5Y, was transfected with GFP-TDP-43 for 48 hours before fractionation. Levels of GFP-TDP-43, phosphorylated-TDP-43 and endogenous TDP-43 are shown (n=3).

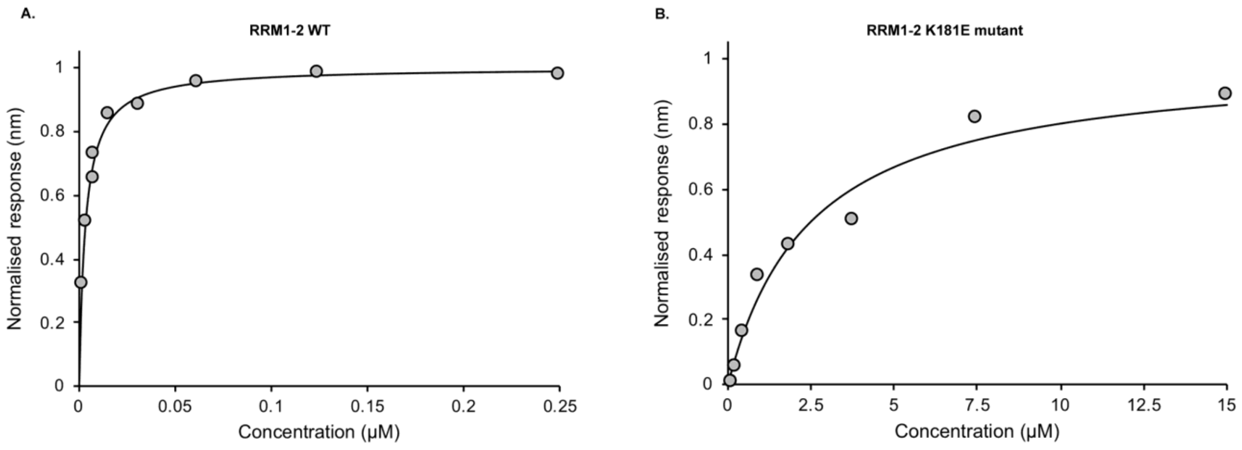

Supplementary Figure 2

Position of K181 and D169 residues in relation to GU RNA. Crystal structure of TDP-43 N-terminal fragment containing two RRM domains (in grey) interacting with a GU-rich RNA (in yellow). K181 and D169 residues are highlighted in blue.

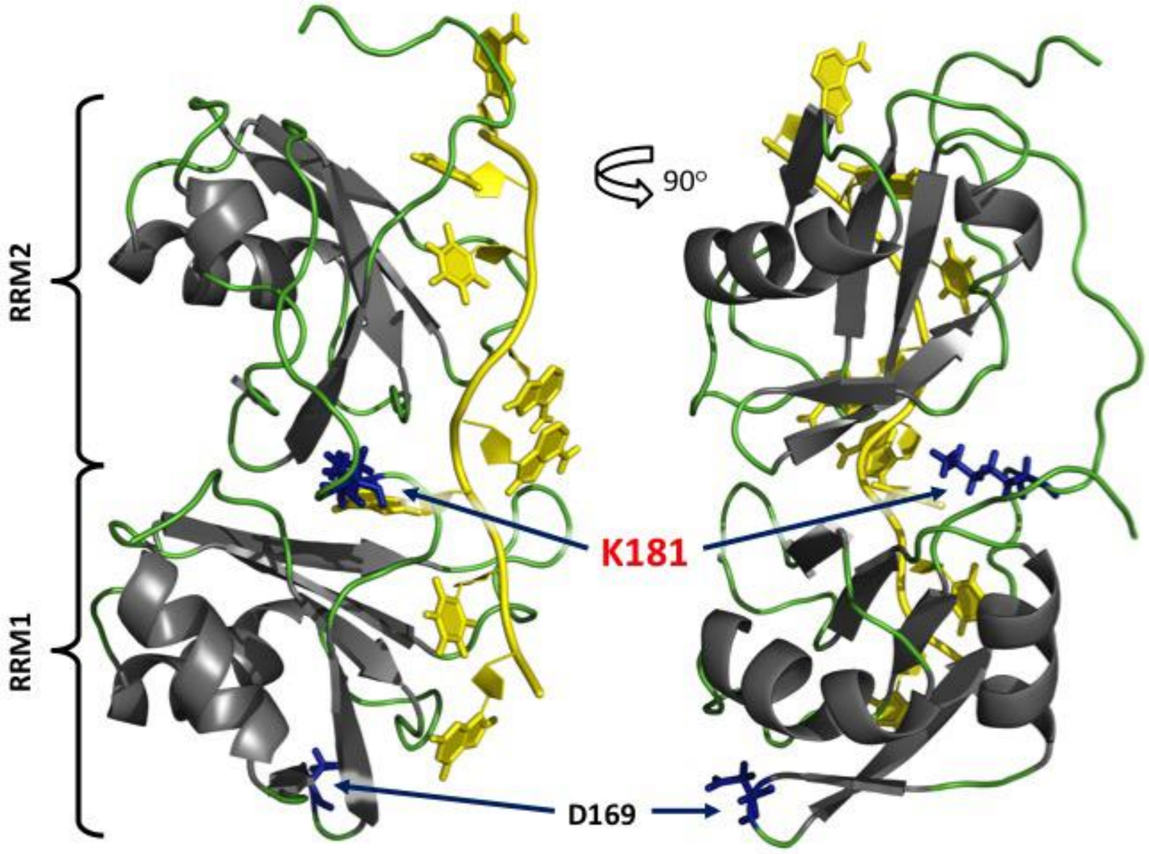

Supplementary Figure 3

Frequencies and locations of nonsynonymous TDP-43 variants in the ExAC database. This diagram shows, from top to bottom: i) Allele counts of nonsynonymous TDP-43 variants in ExAC (blue bars, in base-4 bins); ii) Amino acid sequence ("Shapely" colour scheme); iii) Amino acid scale; iv) Exon structure of the coding region; v) Protein domains.

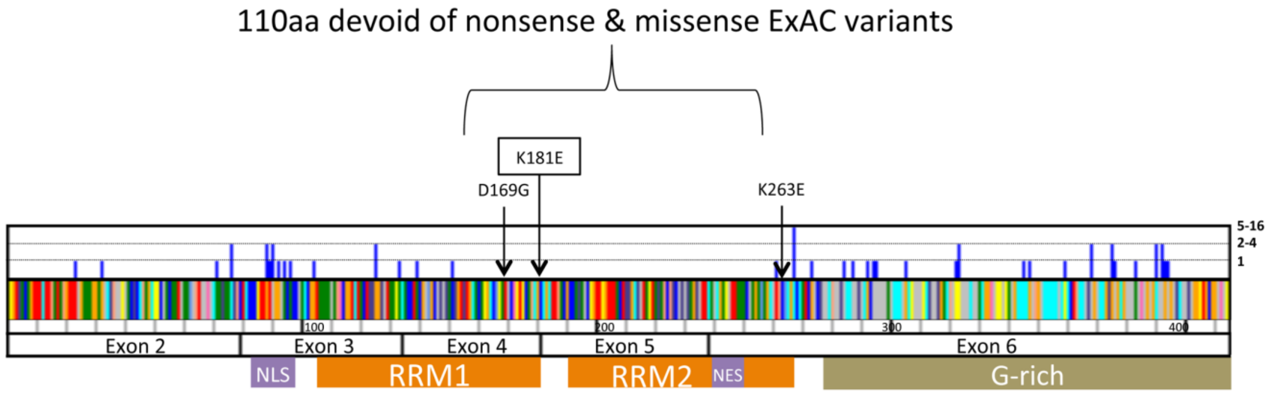

Supplementary Figure 4

Cytoplasmic dNLS/K181E-TDP-43 forms detergent resistant, hyperphosphorylated aggregates in SH-SY5Y cells. (A) SH-SY5Y cells expressing GFP-TDP-43 were harvested and fractionated 24 hours after transfection. Levels of total GFP-TDP-43, phosphorylated TDP-43 and endogenous TDP-43 in the detergent soluble (RIPA) fraction and insoluble (Urea) fraction are shown. Actin in the soluble fraction is used as a loading control. N=3. **(B)** SH-SY5Y cells are fixed and stained for phospho-TDP-43 (red) 48 hours after transfection with GFP-TDP-43 (green). Nuclei are shown with DAPI staining (blue). Scale bars of 10 µm are shown. N=3.

Supplementary Movie 1

Crystal structure of the N-terminal TDP-43 fragment interaction with GU RNA. The N-terminal TDP-43 peptide containing two RRM domains (in yellow) interacting with the GU-RNA is shown (pdb:4bs2). The K181 residue is highlighted in purple.

Supplementary Movie 2

Movie of FRAP on nuclear GFP-WT TDP-43. Photobleaching of nuclear GFP-WT TDP-43 in HEK cells 48 hours after transfection. Fluorescence recovery is recorded for one minute after photobleaching.

Supplementary Movie 3

Movie of FRAP on nuclear GFP-K181E TDP-43. Photobleaching of nuclear GFP-K181E TDP-43 in HEK cells 48 hours after transfection. Fluorescence recovery is recorded for one minute after photobleaching

Supplementary Table 1. Rare variants identified by whole-exome sequencing and shared by both affected members of the ALS/FTD family.

Rare vari	AA change	NOVELTY	Hg19	HGVS	ALSDB	dbSNP 150	UK10K	EVS/ESP	ExAC	GnomAD Genome	Pathogenicity Predictions
ABCC8	A269D	na	chr11:17483146G>T	NM_000352:c.806C>A	0/2630	rs372930264	1/3781	0/6493	0/63352	1/15395	13/20
AFAP1L1	E283K	na	chr5:148689618G>A	NM_152406:c.847G>A	0/2634	rs767387289	0/3781	0/6503	3/63339	0/15395	10/20
ATP7B	M89V	na	chr13:52549091T>C	NM_000053:c.265A>G	0/2468	rs372516400	0/3781	1/6315	1/62212	0/15479	3/20
CD320	T191P	NOVEL	chr19:8367796T>G	NM_016579:c.571A>C	0/2630	rs566090996	0/3781	0/6503	0/63251	0/15479	6/19
CKAP2L	Q16R	NOVEL	chr2:113520137T>C	NM_152515:c.47A>G	0/2636	na	0/3781	0/6503	0/63333	0/15412	0/20
CPNE4	V521I	na	chr3:131261433C>T	NM_153429:c.1561G>A	1/2559	rs779283179	0/3781	0/6503	2/63339	0/15380	15/20
GPR42	G212R	NOVEL	chr19:35862895G>C	NM_001348195:c.634G>C	na	na	0/3781	0/3893	0/22446	0/6312	0/19
IGLON5		na	chr19:51828588C>G	NM_001101372:c.392-12C>G	na	rs755433421	0/3781	0/6328	2/60533	0/15446	3/5 (s/v)
KANK4	E304K	na	chr1:62739866C>T	NM_181712:c.910G>A	1/2612	rs371348159	0/3781	1/6503	2/63314	0/15479	12/20
LSMEM2	R153W	na	chr3:50324595C>T	NM_153215:c.457C>T	0/2462	rs200726517	1/3781	0/6503	1/63225	0/15380	7/20
MAP7	K568K	na	chr6:136681934T>C	NM_003980:c.1704A>G	0/2638	rs772067143	0/3781	0/6503	1/63339	1/15429	1/5 (s/v)
MCM9	I261T	NOVEL	chr6:119238848A>G	NM_153255:c.782T>C	0/2598	na	0/3781	0/6503	0/63333	0/15363	9/20
MELK	K156N	NOVEL	chr9:36597281A>C	NM_014791:c.468A>C	0/2636	na	0/3781	0/6503	0/63339	0/15496	0/20
NKTR	P579S	na	chr3:42678931C>T	NM_005385:c.1735C>T	1/2340	rs764142260	0/3781	0/6503	1/62940	1/15496	5/20
OR10J5	N110Y	na	chr1:159505470T>A	NM_001004469:c.328A>T	1/2577	rs780730919	0/3781	0/6503	1/63352	0/15496	3/19
OR2M4	A119P	na	chr1:248402585G>C	NM_017504:c.355G>C	0/2597	rs377343989	0/3781	1/6503	3/63339	1/15496	11/20
PAPOLB	K33E	NOVEL	chr7:4901345T>C	NM_020144:c.97A>G	na	na	0/3781	0/6098	0/56510	0/15496	3/16
PDZRN4	R54G	na	chr12:41900348A>G	NM_013377:c.160A>G	0/2374	rs753561493	0/3781	0/6503	2/63276	0/15496	16/20
PSTPIP1	H234Q	NOVEL	chr15:77323580C>G	NM_003978:c.702C>G	0/2560	na	0/3781	0/6354	0/61806	0/15380	7/20
PTBP1	I30M	na	chr19:803611C>G	NM_031991:c.90C>G	0/2638	rs757290221	1/3781	0/6503	3/63314	0/15446	10/20
RREB1	S98A	NOVEL	chr6:7189422T>G	NM_001168344:c.292T>G	0/2441	na	0/3781	0/6503	0/63168	0/15380	4/20
RYR3		NOVEL	chr15:34015098T>A	NM_001036:c.6800+2T>A	0/2583	na	0/3781	0/6029	0/60907	0/15496	5/5 (s/v)
SP7	K230R	na	chr12:53722537T>C	NM_152860:c.689A>G	0/2620	rs376163810	0/3781	1/6029	1/61743	1/15479	8/20
TANGO6	I356V	na	chr16:68909128A>G	NM_024562:c.1066A>G	0/2637	na	0/3781	0/6333	0/61958	1/15429	13/19
**TARDBP**	**K181E**	**NOVEL**	**chr1:11078928A>G**	**NM_007375:c.541A>G**	**0/2432**	**na**	**0/3781**	**0/6503**	**0/63010**	**0/15412**	**15/20**
TAX1BP1	I238T	na	chr7:27824882T>C	NM_006024:c.713T>C	0/2580	rs755060245	0/3781	0/6503	2/63282	1/15446	13/20
TBC1D4	T625M	NOVEL	chr13:75900492G>A	NM_014832:c.1874C>T	0/2550	na	0/3781	0/6206	0/62193	0/15479	6/20
USP32	S1127F	na	chr17:58275675G>A	NM_032582:c.3380C>T	0/2616	na	0/3781	0/6503	0/63352	1/15462	16/20
WDR75	N786T	na	chr2:190340007A>C	NM_032168:c.2357A>C	0/2616	rs753990418	0/3781	0/6501	1/63048	0/15462	3/20
